# CNest: A novel copy number association discovery method uncovers 862 new associations from 200,629 whole-exome sequence datasets in the UK Biobank

**DOI:** 10.1016/j.xgen.2022.100167

**Published:** 2022-08-10

**Authors:** Tomas Fitzgerald, Ewan Birney

**Affiliations:** 1 European Molecular Biology Laboratory, European Bioinformatics Institute (EMBL-EBI), Wellcome Genome Campus, Cambridge CB10 1SD, UK

**Keywords:** copy number variation, whole-exome sequencing, next-generation sequencing, genome-wide association studies

## Abstract

Copy number variation (CNV) is known to influence human traits, having a rich history of research into common and rare genetic disease, and although CNV is accepted as an important class of genomic variation, progress on copy-number-based genome-wide association studies (GWASs) from next-generation sequencing (NGS) data has been limited. Here we present a novel method for large-scale copy number analysis from NGS data generating robust copy number estimates and allowing copy number GWASs (CN-GWASs) to be performed genome-wide in discovery mode. We provide a detailed analysis in the UK Biobank resource and a specifically designed software package. We use these methods to perform CN-GWAS analysis across 78 human traits, discovering over 800 genetic associations that are likely to contribute strongly to trait distributions. Finally, we compare CNV and SNP association signals across the same traits and samples, defining specific CNV association classes.

## Highlights

• Novel copy number variation (CNV) associations in a large human cohort
• Large-scale CNV association testing from next-generation sequencing data
• Specifically designed GWAS framework for CNV operating under GA4GH standards
• Investigation of SNP and CNV associations and CNV tagging SNPs

## Graphical abstract

**Figure:**
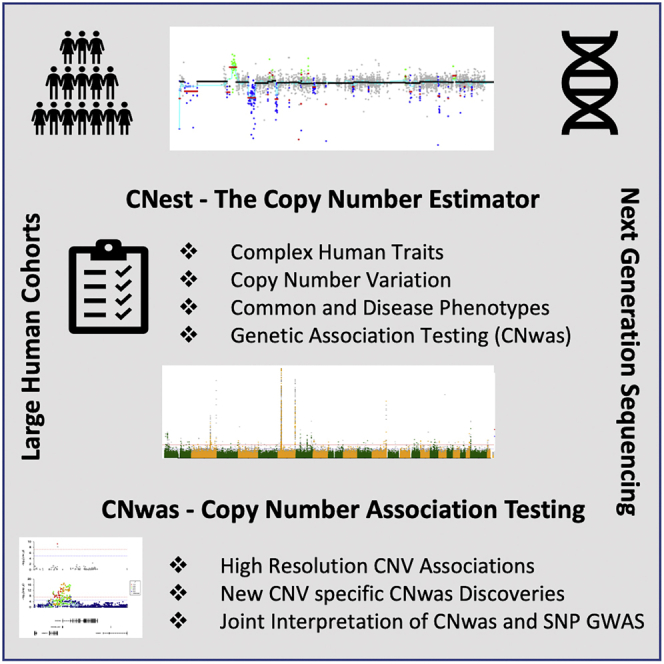

## Teaser

CNest copy number variation (CNV) genome-wide association study (GWAS) testing brings CNVs into a similar framework as those widely used in SNP GWAS. We made over 800 new discoveries in the UK Biobank human cohort at exon-level resolution, paving the way for further CNV GWAS studies using next-generation sequencing data.

## Introduction

Genome-wide association studies (GWASs) are a well-established genetic technique, having made thousands of robust associations between traits and sequence-level genetic variation.^1^, ^2^, ^3^, ^4^, ^5^, ^6^, ^7^ Often these associations can have significant impacts on the understanding and, in some cases, the treatment of human disease.^8^, ^9^, ^10^ However, for most common genetic diseases, these associations only account for part of the heritable disease risk.^11^, ^12^, ^13^ In terms of total base pairs, copy number variation (CNV) accounts for the majority of differences between any two genomes^14^, ^15^, ^16^, ^17^, ^18^ and is known to alter human trait distributions,^19^, ^20^, ^21^ often with a strong impact on human health.^22^^,^^23^ This is highlighted best within the large body of research studying CNV in relation to rare genetic disease.^24^, ^25^, ^26^ Although it is widely accepted that CNV can contribute significantly to differences in human traits, to date, methods for large-scale CNV to phenotype association studies, the equivalent of GWAS for CNVs, have been hampered by a number of factors, including methodological difficulties,^27^ the availability of sufficiently large datasets, and the ability to interpret complex rearrangements from sequencing data.^28^^,^^29^

CNVs have been a major component of routine clinical medical genetics screening for over a decade; however, the interpretation of individual events remains challenging,^30^^,^^31^ with most clinical testing laboratories routinely finding potentially pathogenic CNVs in patients with intellectual disability, autism spectrum disorders, and/or multiple congenital anomalies.^32^, ^33^, ^34^ Although CNV detection from sequence data in a clinical setting is in active development, most clinical CNVs are still discovered using specialized microarrays.^35^ Most CNVs with strong effects are rare and they are often discovered as *de novo* mutations in patients across a range of genomic disorders.^36^^,^^37^ Furthermore, it has been observed that the overall burden of CNVs is higher in specific patient groups compared with controls^38^^,^^39^ indicating potential combinatorial CNV effects.^40^ It is conceivable that specific combinations of CNVs, by acting in concert, may have a large potential to cause phenotypic differences due to factors such as dosage compensation, incomplete penetrance, and polygenic effects.^41^^,^^42^ The impact of CNVs in rare diseases is likely to be large, whereas one might expect weaker effects of all variation in more common diseases, consistent with the polygenic behavior of these diseases.

Several CNV genotype-phenotype correlations have been observed in relatively small-scale studies of specific patient groups^43^ or by collaborative efforts to share genetic data for rare disease;^44^ however, CNVs have also been associated with a number of complex diseases.^45^^,^^46^ Recent large-scale CNV association testing using datasets such as the UK Biobank have found some highly significant loci in relation to certain human traits,^47^ and previous studies focused on cognitive traits such as schizophrenia^48^ and autism^49^ have demonstrated the utility of SNP arrays to search for novel CNV associations. Focused studies into specific human traits have used large-scale SNP genotypes to perform association testing with great success;^50^, ^51^, ^52^, ^53^, ^54^ however, these studies have often focused on predefined lists of CNV regions known to be important within a clinical setting.^53^ Another important consideration is that SNP genotyping arrays have a limited resolution to detect small CNVs and a limited sensitivity for CNV discovery genome-wide due to the distribution of SNPs across the genome and a limited dose response.^55^ A recent CNV association study showed both the power and limitations of genotype-based CNV association testing in the UK Biobank, finding 131 significant signals across 47 quantitative traits.^56^ The smallest CNV association detected was 49 kb at 1p36.11 found to be in association with reticulocyte count, platelet count, and hemoglobin A1c (HbA1c).^56^ However, most of the CNV association signals detected involved large recurrent CNVs with a mean size of 817 kb, highlighting the limited resolution when using SNP arrays. Another recent study showed an improvement in resolution from SNP arrays by including information on shared extended SNP haplotypes into their model, detecting 269 independent associations across 56 quantitative traits.^57^ Both studies tested quantitative traits only and were limited to the resolution of the SNP array; nevertheless, both found novel discoveries, highlighting a large potential for CNV association testing genome-wide for complex human traits.

It is reasonable to assume that CNVs may account for a substantial portion of the variance observed in common disease risk. Some of these CNVs will be in strong linkage disequilibrium (LD) with SNPs, and so they can be discovered by tagging polymorphisms, but the causal change is impossible to narrow down using SNPs alone. Other CNVs might not have good tagging SNPs and, furthermore, recurrent CNVs are far more common than recurrent SNPs, with the CNV mutation rate currently estimated at 0.2 *de novo* events per individual compared with between 1.8× 10⁻^8^ and 2.5 × 10⁻⁸ per base pair per generation for point mutations,^19^^,^^58^, ^59^, ^60^ meaning that the aggregate higher-frequency CNVs with the same functional impact are hard to model using the combination of rare haplotypes. With the advance of large data cohorts with datasets that are amenable to copy number estimation,^61^, ^62^, ^63^ the ability to perform high-resolution genome-wide GWAS testing for CNVs has become more feasible. One challenge for large-scale CNV discovery has been variability in raw sequencing depth due to other factors, most likely extraction techniques and immune system state at the time of blood draw. This variation gives rise to complex noise characteristics in raw sequencing read depth between samples, so called genomic waves. To explore this, one needs robust normalization strategies for CNVs, an appropriate discovery method for CNVs, and a way to easily integrate both CNV- and SNP-based associations into one framework.

In this work, we address some of these issues by providing a new discovery method for CNVs from next-generation sequencing (NGS) data, CNest, based on novel normalization techniques for large-scale cohorts. Rather than trying to create individual models of alleles for each CNV locus, we have chosen to use a straightforward linear model for discovery. This linear model is both consistent across all CNV loci and has many similar properties to the linear models used in SNP GWAS. As such, we can use the same covariates, the same diagnostic style QQ plots, and place SNPs and CNVs associations into the same framework. Post discovery, we show we can provide more detailed modeling of at least some loci. We provide a comprehensive CNV analysis using this method on the large UK Biobank cohort with exome sequences. To explore the relationship with established SNP polymorphisms, we also performed both CNV and SNP GWAS within a single framework, applying our methods across the same set of UK Biobank samples and interrogating the resulting associations across a diverse set of traits.

We find many CNV to phenotype associations, although, as expected, many of these associations are also tagged by SNP polymorphisms. However, we have a subset of CNV associations that cannot be discovered via SNPs and another subset that are coincident with strong SNP polymorphisms but not well correlated with any specific SNP, and many where the CNV is taggable but the tagged SNP is at some distance from the CNV locus. Many of these associations recapitulate multiple known associations based on previous studies on both CNV and SNP genome association testing, whereas others discover new CNV-specific findings in relation to the genetics of common human traits. We have made the software, CNest, which performs this discovery open source and provided portable workflows to run CNest, compatible with GA4GH standards.^64^

## Results

### CNV in 200,629 individuals from the UK Biobank

To identify exon-resolution CNV regions across a large population of individuals from NGS data in the UK Biobank, we developed a suite of flexible, highly scalable CNV analysis tools known as CNest (see STAR Methods). Within this package, we include a robust CNV caller as well as a set of tools and novel approaches to CNV association testing genome-wide in discovery mode. A flow diagram describing the major steps performed for CNest calling and association testing can be found in Figure S1. A central component of these methods is the selection of appropriate reference datasets and normalization procedures by modeling certain noise characteristics of whole-exome sequencing (WES)/whole-genome sequencing (WGS), for example, the presence and scale of genomic waves, to generate optimized copy number measurements across large sample cohorts (see STAR Methods). After calling CNVs in the 200,629 sample cohort with WES data, we applied several quality control (QC) measures to ensure that the copy number measurements and CNV calls were consistent.

**Figure 1:**
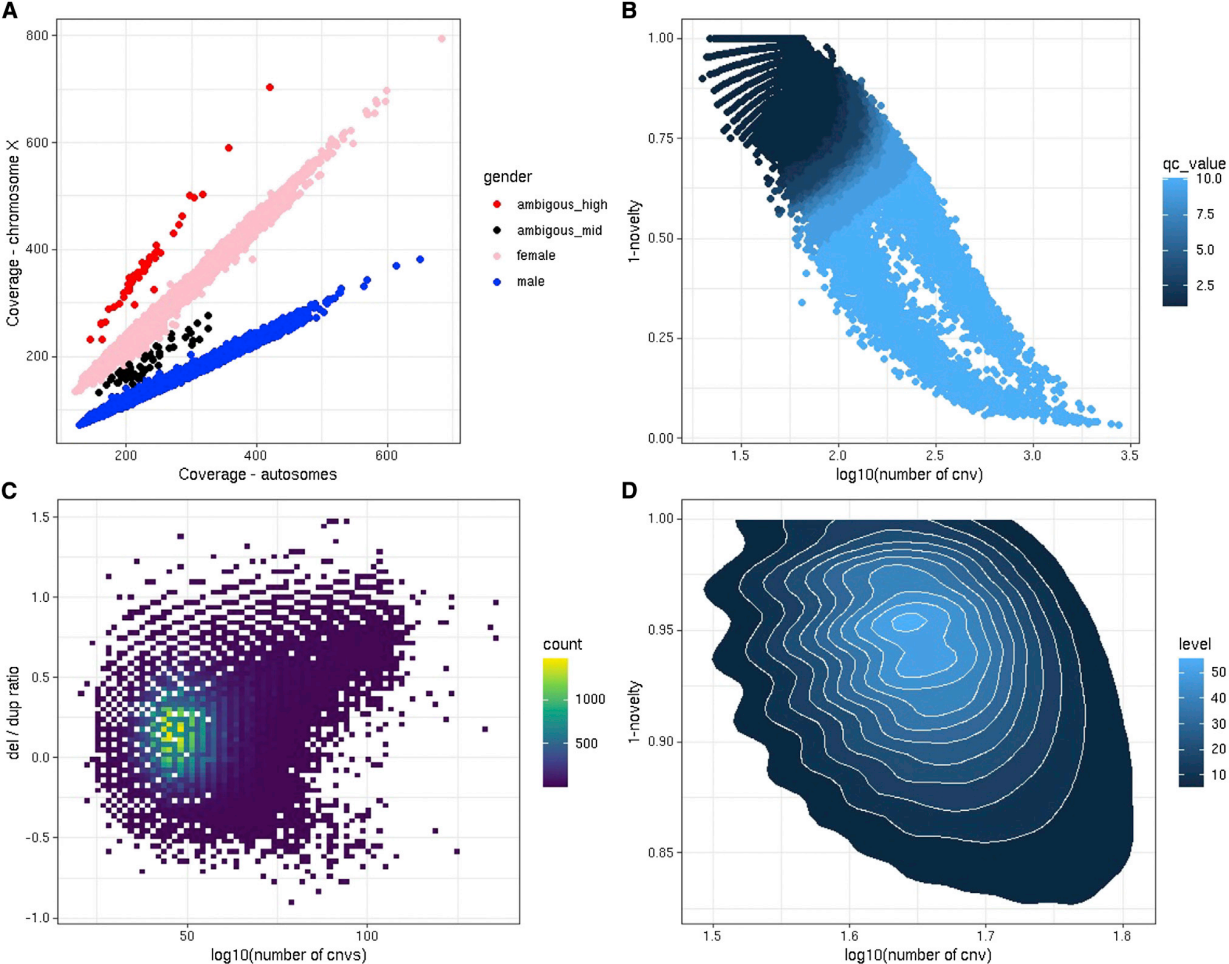
QC of CNV calls in the 200,629 UK Biobank exome sequences (A) Gender classification, the relative coverage of autosomes compared with chromosome X and the CNest gender classifications shown in different colors across all samples. (B) The total number of autosomal CNV calls versus a measure of the proportion of rare CNVs per sample using a 1% population frequency. (C) The log10 of the loss to gain ratio versus log10 of the total number of CNV calls for each sample. (D) A density plot showing (B) but for QC-passed samples only.

A subset of the diagnostic plots of CNest is shown in Figure 1. An obvious but important step in CNV analysis is the classification of sex based on the estimated copy number of the X chromosome. Well-controlled one versus two copy number of the X chromosome indicates that the normalization procedure and relative copy number estimates have worked successfully, at least for the X chromosome (Figure 1A). A side effect of this analysis is the ability to detect sex chromosome aneuploidy. We detected 50 samples showing an unusually high number of copies on chromosome X (Figure 1A). These samples were assumed to be a mixture of data quality issues and real triple X cases. Triple X is a condition caused by random error during reproductive cell division and is found in approximately one in 1,000 women. Although triple X has been associated with several trait differences, it can often go undiagnosed and, depending on other social factors, may never give rise to any noticeable problems.^65^ We also detected 51 datasets that show an unusual level of chromosome X coverage and cannot be reliably assigned to either double (female) or single X (male). These we assume to be both inconsistent capture of chromosomal baits and potential mosaic sex chromosome events (i.e., mosaic XXY).

Like previous studies into sex chromosome aneuploidy in the UK Biobank,^66^ we classify 50 out of 110,312 women as potential trisomy X (Ambigous_high) giving a prevalence of 45.3 out of 100,000. We also compared the sex classification made by the SNP arrays (f22001), the prediction of sex chromosome aneuploidy (f22019), and the presence of any International Classification of Diseases, Tenth Revision (ICD10) code for sex chromosome abnormality, Q90 to Q99 (Table S1). The majority of sex classifications agree between the exome sequence and SNP array data, and those that are discordant are enriched for the presence of both f22019 and sex chromosome abnormality-related ICD10 codes. Sex chromosome aneuploidy is not a focus of this study, and we simply exclude all samples that could not be reliably assigned to either double or single X based on their coverage profiles (Figure 1A); these copy number sex chromosome calls will be returned to the UK Biobank for further investigation by other investigators.

Some informative CNV quality information is contained within the consistency in the number of CNV calls in all samples against the proportion of those calls that are rare across the entire population (Figure 1B). This is like the genotyping extreme heterozygosity quality parameter used as standard in SNP genotyping QC. Given current estimates on the CNV mutation rate,^67^ we would expect very low numbers of *de novo* CNV events (less than one per genome) and rare CNVs to be infrequent in any individual genome, which is supported empirically here with a median of three rare CNVs per UK Biobank exome based on a 1% population frequency for losses and gains separately. For large-scale CNV analysis in assumed healthy individuals, it is sensible to assume that most genomes will on average display a consistent level of rare variation compared with the bulk of the population. Encouragingly, after applying our strictest definition of QC across greater than 200,000 exome sequences for CNV calling, we obtain a greater than 92% pass rate, indicating that, for most samples, our CNV estimation and calling approach is consistent. There is no reason to expect, given known CNV formation mechanisms such as non-allelic homologous recombination (NAHR) and non-homologous end-joining (NHEJ), that there would be any bias between the number of losses and gains when comparing large numbers of genomes in aggregate, and although there are some outliers, we observe a tight loss-to-gain ratio distribution with a median of 1.4 (Figure 1C). When assessing these distributions in samples that passed our QC criteria, the bulk of the data are tightly centered around a mean number of calls of 48 and a mean rarity rate of 0.07 (Figure 1D).

As expected, we observe a bias in loss/gain detection with a median of 28 losses compared with 19 gains per sample (Figure S2A); most CNV callers from both array- and sequence-based data show an increased ability to detect losses compared with gains due to the increased variance for higher copy number signals (“reads”) and a lowered dose response.^68^, ^69^, ^70^ This decreased dose response makes it more challenging to detect gains, often requiring a larger number of consistent signals (“responding probes”) to be able to distinguish real signals from baseline noise properties.^71^^,^^72^ When looking at the proportion of CNV calls (deletions and duplications) made by CNest across all 200,000 UK Biobank samples, most calls are small (51% of all calls <100 kb) and the difference in loss-to-gain sensitivity is most evident for smaller events (Figure S2B). Most CNVs detected are small; however, only 1.8% of all calls include only a single exon, and, as size increases, the proportion of losses to gains stabilizes to approximately equal numbers above a size of 500 kb, with calls above this size accounting for 14.1% of the total call set (Figure S2B). Common CNVs are not uniformly distributed throughout the genome, and we find several high-frequency recurrent events in known CNV formation hotspots (often closer to low copy repeats and centromeric regions). We see strong correlation between the number of CNVs called by CNest for each chromosome with the total number of annotated segmental duplications for that chromosome (Figure S2C). There is a stronger correlation for losses compared with gains (Pearson’s *R* of 0.82 for losses compared with 0.75 for gains), which is likely due to the dose response difference and decreased sensitivity for smaller gains. To assess the presence of the genomic wave within our final log2 ratio distribution, we calculated a genome wave estimate based on the interquartile range (IQR) of a running median using a 401-data-point span scaled by a scaling factor. Across all 200,000 sample-level normalized log2 ratio distributions, we observe very low levels of extreme wave characteristics, with only 3.2% of all samples having a genomic wave estimate greater than 1 (Figure S2D). Samples with a wave estimate greater than one represent those for which we would expect that the presence of wave-based noise in their log2 ratio distribution may make CNV calling challenging (Figure S3). When looking at the wave estimates in relation to the total number of losses and gains made per sample, we observe very tight distributions across the full range of total CNV calls (Figure S2D). Interestingly, our sample-level CNV calls appear to be largely robust to differences in wave-based noise, with most samples showing higher wave estimates being within the lower ranges of total CNV calls.

To further assess some characteristics of our CNV calls, we looked at how many predicted loss-of-function CNVs (either deletion or truncating duplications) overlapped clinically important genes from the dd gene to phenotype (DDG2P) resource;^24^ we expect the common CNVs discovered in UK Biobank to be depleted in overlaps to these genes. Using a 50% reciprocal overlap rule against 218 mono-allelic loss-of-function genes from the DDG2P, we found a total of 342 individuals CNV calls (Figure S4), 40% of which were in the same gene, *GLMN*, which is known to be involved in glomuvenous malformations.^73^ Overall, similar to previous work on pathogenic CNVs in the UK Biobank,^52^ we detect small numbers of CNVs in clinically important disease genes across the UK Biobank, and rare variant analysis is not a focus of this study; however, we encourage interested researchers to make use of these high-resolution CNV calls (see data and code availability) where it might be possible to look at modifier effects for rare CNV events.

### Copy number variation association testing in the UK Biobank

For CNV association testing genome-wide in discovery mode, we made use of both the copy number estimates and CNV calls generated by CNest and applied standard linear and logistic regression models using the copy number estimates as CNV dosage (see STAR Methods), analogous to the common dosage model of alleles from SNPs. Although the choice of linear models restricts our signal to sites displaying a linear relationship between copy number and trait, more sophisticated models that could have non-linear impacts on phenotypes can be complex to select and even more complex to analyze the resulting statistics consistently genome-wide. Furthermore, this simple model is like those most often used in SNP GWAS^74^ and so is more easily jointly integrated with SNP discovery. All models were applied to unrelated samples from the principal-component analysis (PCA)-defined European cluster (SNP principal components [PCs] 1 and 2) and include standard covariates with 10 PCs derived from both SNP and CNV estimates independently.

**Figure 2:**
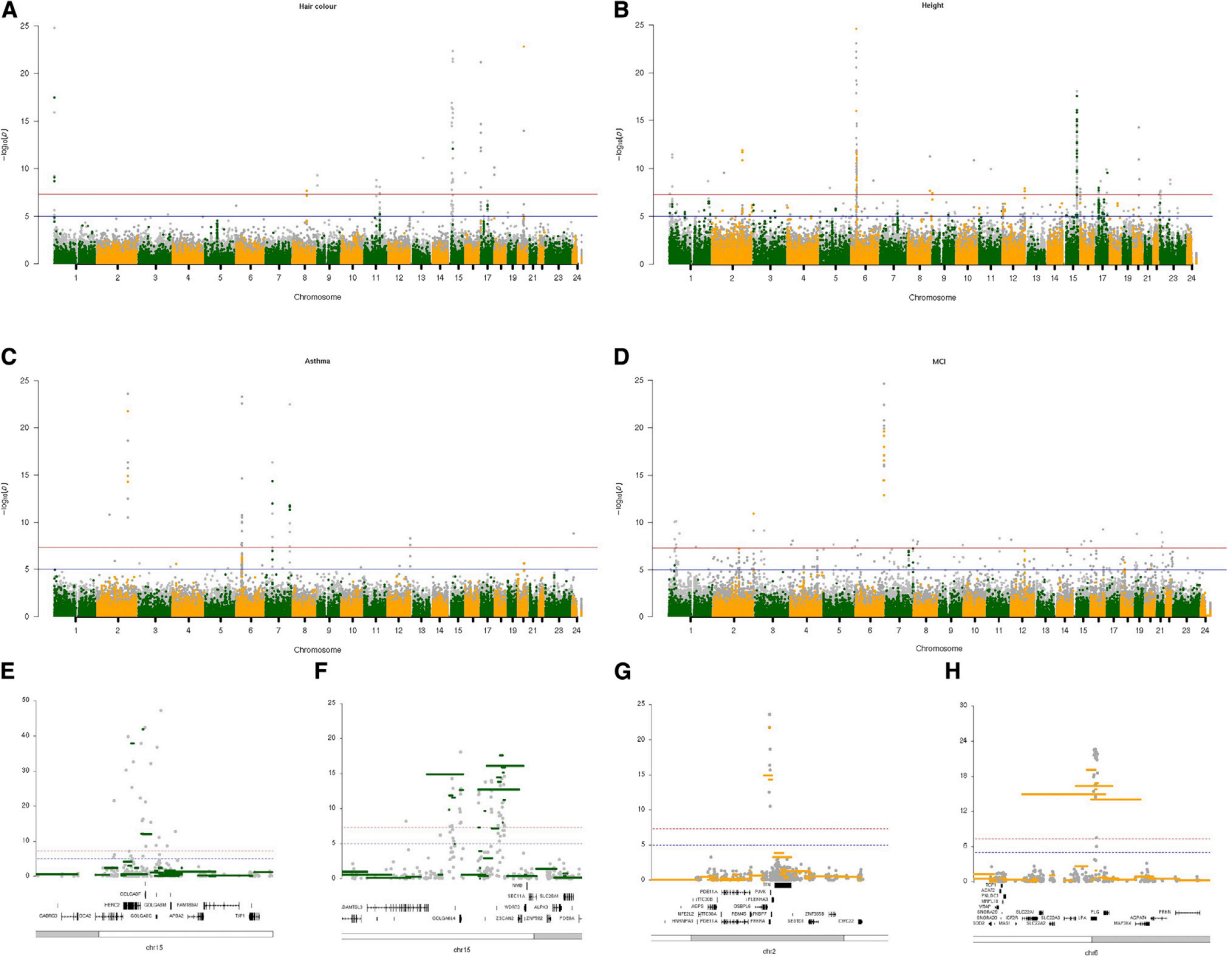
Copy number association Manhattan plots for four different UK Biobank traits Exon-level signals are shown in different shades of gray and CNV call level signals in orange and green. (A–D) Associations for (A) hair color using a linear model, (B) associations for standing height using a linear model, (C) associations for disease coding asthma using a logistic model, (D) associations for disease coding myocardial infarction using a logistic model. (E) Zoom locus plot showing chr15 around the OCA2/HERC2 genes for hair color signal. (F) Zoom locus plot showing chr15 around the ADAMTSL3/UBE2Q2L/GOLGA6L4 genes for standing height signal. (G) Zoom locus plot showing chr2 around the genes CHROMR, PRKRA, and PJVK for asthma signal. (H) Zoom locus plot showing chr6 around the LPA gene for myocardial infarction signal.

We performed CNV association testing for 46 different main UK Biobank fields, including 30 quantitative and 16 binary traits across a variety of physiological, lifestyle, and health-related categories (Table S2). We used diagnostic QQ plots and the associated genomic inflation statistic to be confident that our model produced a well-behaved statistical test in which the majority of the genome fits the expected null hypothesis (Table S2). In total, after fine mapping to select the most associated probe for each CNV-phenotype association at a locus (see STAR Methods) we discovered 646 significant CNV-specific associations across 34 traits, 24 quantitative and 10 binary (Figure S5). We also selected all instances of the First Occurrences UK Biobank field that had greater than 500 cases mapping to an ICD10 code (UK Biobank field 1712), resulting in 398 different codes that we used as case/control labels for CNV association testing with logistic regression models. These 398 labels covered 15 broader categories (Table S3), and we obtained significant associations for 44 ICD10 codes across 13 broad categories. We show some specific examples (Figure 2) to illustrate these associations and their concordance to previous studies (see Data S1 for a further description).

For most UK Biobank main traits tested, we discovered new CNV-specific associations (Table S8); for example, for the eye-related trait corneal hysteresis, we detect robust CNV associations in exons 15 to 36 of the *ANAPC1* gene (Figure S6), in which sequence variation has been estimated to account for 24% of corneal endothelial cell density variability;^75^ and for both corneal hysteresis and intraocular pressure, we discover exon-level associations in the important *TCF4* gene, which is known to be involved in several eye disorders, such as Fuchs corneal dystrophy^76^ as well as haploinsufficiency of *TCF4* being strongly associated with Pitt-Hopkins syndrome.^77^ Given that the UK Biobank participants were broadly healthy at recruitment, this association with eye phenotypes at the *TCF4* locus deserves further investigation. For red blood cell-related traits we detect a large number of associations that have prior evidence of association from SNP GWAS (Figure S7), such as variation in and around the *ABO* gene;^78^ for lifestyle measures such as alcohol consumption, we find associations within known genes^79^ such as *NPIPB6*; and for cognitive measures, we also discover CNV association in genes with previous evidence of association from SNP GWAS testing in the UK Biobank, such as the *ARL17B* gene in association with reaction speed.^80^

All these CNV discoveries deserve integration with SNP polymorphisms and the often well-studied biology around these loci, and, as described in the “data and code availability” section, we have made all these results available to the community in a variety of ways. Here we provide important insight into the type of results possible to achieve for copy number association testing in large NGS cohorts bringing CNV GWAS into a similar framework to SNP-based tests and paving the way for further extensive studies to investigate the relationship between copy number and complex traits in humans.

### First occurrences ICD10 code CNV associations

**Figure 3:**
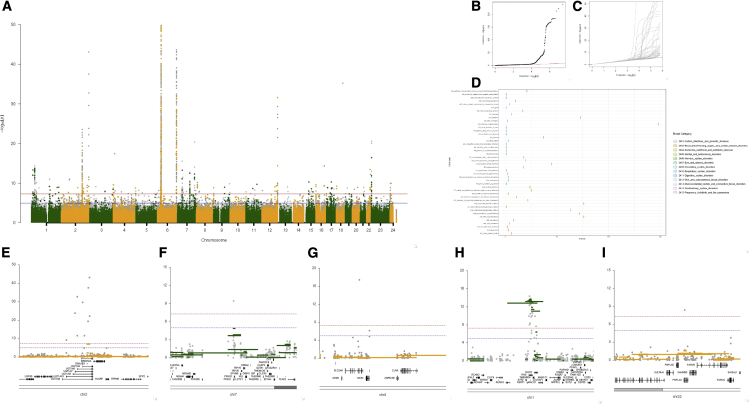
ICD10 code case/control copy number associations (A) Combined and overlaid Manhattan plot for CNV associations across 44 ICD10 codes. (B) Combined QQ plot including all p values from association results across all 44 traits. (C) Overlaid QQ plots showing all individual QQ plots for the 44 traits. (D) Plot showing the total number of exons for all ICD10 codes that had any significant signal. (E) Locus zoom plot at UGT1A genes for ICD10 code E80 (disorders of porphyrin and bilirubin metabolism). (F) Locus zoom plot at the PRSS1 gene for ICD10 code D50 (iron deficiency anemia). (G) Locus zoom plot at the SLC2A9 gene for ICD10 code M10 (gout). (H) Locus zoom plot at the RHD and RHCE genes for ICD10 code O 36 (maternal care for known or suspected fetal problems). (I) Locus zoom plot at the PNPLA3 gene for ICD10 code K74 (fibrosis and cirrhosis of liver).

To complement the UK Biobank measured and binary traits we also explore CNV associations to direct healthcare measures, as represented by the Hospital Episode Statistics (HES)-captured data on ICD10 codes in the UK Biobank. We performed CNV association testing using the First Occurrences fields as case control labels for all codes that had greater than 500 cases and did not preselect or filter out any case labels, running CNV association testing across a total of 398 case control labels (Table S3). Across all 398 codes, we discovered 242 CNV-specific associations across 44 codes covering 144 unique genes (Figure 3A). A large fraction (117 out of 242) were located within the human leukocyte antigen (HLA) super locus at 6q21 between chromosome positions chr6:30500001 and 46200000, and there were six traits that had no associations outside of the HLA super locus, 13 traits that had associations both within and outside of the HLA, and 25 traits that had associations exclusively outside of the HLA.

After fine mapping, the majority of ICD10 codes (27 out of 44) had between one and two significantly associating regions, with nine ICD10 codes having between three and 10, and eight ICD10 codes having greater than 10 associations. Almost all the association results were well controlled with inflation factors (lambda) ranging from 0.984 to 1.140, with the exception of ICD10 code F17, mental and behavioral disorders due to use of tobacco, which showed mild inflation with a lambda of 1.382 (Figures 3B and 3C; Table S2). Most fine-mapped regions were small (Table S9), with a median number of significant exons of three per fine-mapped region (Figure 3D), with the largest region involving 52 exons across five different genes in association with ICD10 code K90: intestinal malabsorption. In total, we detected 242 associations ranging from well-known important regions of the genome through to completely novel findings based on CNVs alone. All association results for the 44 significantly associating ICD10 codes are provided in the supplemental information. We provide specific examples (Figure 3) showcasing some of the new CNV associations that we have made by describing in detail some of the associations discovered across 10 different ICD10 code case control sets (see Data S2 for a further description).

In summary, we discovered 862 new fine-mapped CNV associations across 78 different traits (24 quantitative and 54 binary) using the 200,000 whole-exome release from the UK Biobank, the majority of which have either been previously discovered by SNP GWAS tests or have compelling evidence from other research areas such as health care, rare disease, or animal models (supplemental information), but a significant minority are entirely novel. We were able to detect more associations on average from quantitative compared with binary traits, with a median of four associations per quantitative trait compared with two for binary traits. These new association results and genome-wide association testing approach for CNV provides important insights into the contribution of CNV in complex human traits, which in some cases can have a direct relevance to health-related outcomes and genetic risk profiles. We encourage interested readers to pursue the discoveries discussed here and listed in our supplemental information.

### Comparison with recent CNV association studies using SNP genotyping arrays

There have been some recent studies leveraging the ability of SNP genotyping arrays to detect CNVs and perform genome-wide association testing for copy number.^56^^,^^57^ Although the technology is different and both recent studies used all available UK Biobank participants (∼450,000), we sought to compare the associations we obtained here against those that could be detected using SNP arrays in the UK Biobank. Overall Auwerx et al.^56^ detected 131 new associations across 47 quantitative traits with a mean size of 715 kb, and Hujoei et al.,^57^ who additionally utilized identity by decent (IBD) information into the CNV detection, found 269 associations across 56 quantitative traits with a mean size of 467 kb compared with 862 associations across 78 quantitative and binary traits with a mean size of 9,970 bp with CNest using WES (Figures S9A and S9B).

Both recent SNP-based studies only performed GWAS on quantitative traits including many blood-related measurements and metabolic traits and there were nine traits overlapping both SNP array studies and the CNest results, resulting in 57 CNV associations that we could compare (Table S10). To compare these associations, we remapped all SNP array-based associations to the latest genome build (GRCh38) and interrogated the exome association signals across each site from the association tests on the same trait (see STAR Methods). In total, we found that 63% (36 out of 57) of locations could be confirmed (19 genome-wide and 17 suggestive) across all traits (Figure S9C), and certain traits (e.g., height and reticulocyte counts) had higher levels of agreement (Figure S9D). Some signals only reached suggestive significance levels, and we expect these would likely increase to genome-wide levels with larger sample sizes, whereas some regions showed no evidence of association from CNest association testing (Figures S9E–S9G). We also performed 100 rounds of permutation for each association (see STAR Methods) showing that the number of suggestive signals found for each trait was significantly higher (p = 0.018) for the SNP array-based CNV association regions compared with random areas of the genome with the same size (Table S11). This is a robust but by no means perfect concordance of CNV associations from these very different datasets (SNP based and WES based), different sample sizes, and different detailed methods (how the association is modeled). As expected, using WES, CNest results achieved higher resolution, detecting many smaller associations than both SNP-based studies using less than half the number of samples, variation exclusively in coding regions (exons) and the standard additive model most often applied to SNP GWASs. As all studies were able to find some unique associations, it may be useful to combine copy number information from whole-exome and SNP genotyping arrays for future association studies. Furthermore, the SNP array associations that show no signal from CNest results deserve further exploration once results are available using the same samples, traits, and model setups.

### Combined CNV- and SNP-based associations in the UK Biobank

To investigate the relationship between SNP and CNV associations, we ran SNP-based GWAS tests across six quantitative traits using the same samples we used for CNV association testing (see STAR Methods). These six traits were selected to include a range of signals across different regions of the genome with differential signal strengths. The intention is to allow a direct comparison of SNP with CNV association signals across a variety of human traits. This allowed us to start to explore the underlying genome landscape for associations and to classify individual associations into those that were detectable by SNP and CNV GWAS independently against those that are specific to CNVs (see STAR Methods). We classified CNV signals into CNV only (signals that were detectable by CNV GWAS only), CNV-allele (signals that were present at the same locus by both SNP and CNV GWAS but with very little correlation between them), SNP-CNV near (signals that were detectable by both SNP and CNV GWAS and where those signals were highly likely to be assigned to the same gene), and SNP-CNV far (signals that could be detected by both SNP and CNV GWAS but were highly likely to be assigned to different genes).

Across 133 fine-mapped CNV association regions, we classified 17% (23/113) as CNV only, 44% (59 out of 133) as CNV-allele, 28% (38 out of 133) as SNP-CNV near, and 11% (13 out of 133) as SNP-CNV far (Table S12). We choose to be strict in the definition of novel CNV events (CNV only and CNV-allele) by setting the r^2^ cutoff relatively low since very strong SNP-CNV tagging is rare genome-wide. Most exonic signals that could be well tagged by SNPs were found in regions involving recurrent CNVs with 68% found in CNV regions present in greater than 1,854 individuals (1% population frequency). We consider the SNP-CNV-far, CNV-allele, and CNV-only association classes as different types of novel CNV associations, whereas, for SNP-CNV near, we assume that the signals from both variant types are likely to be tagging the same functional variant; nevertheless, the CNV association may well provide functional insight for the locus.

**Figure 4:**
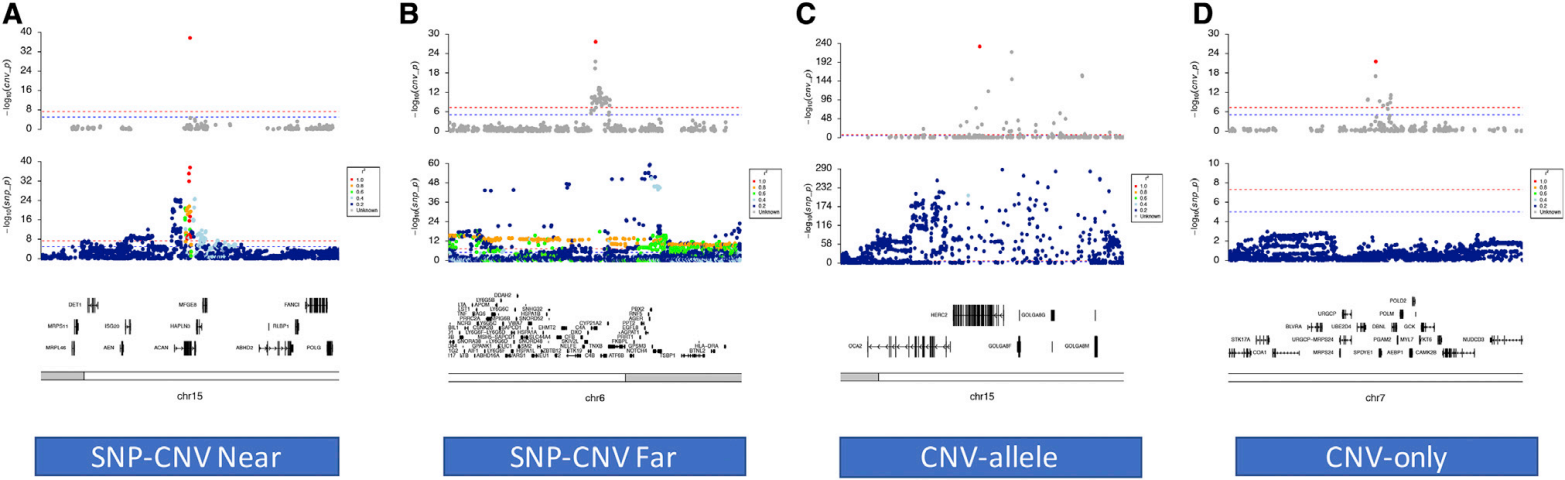
Locus zoom plots showing SNP and CNV association results for the different CNV association type classifications for four different quantitative traits (A) SNP-CNV near association plot for standing height at ACAN. (B) SNP-CNV far association plot for FEV/FEC ratio at C4A. (C) CNV-allele association plot for hair color at HERC2. (D) CNV-only association plot for chronotype at SPDYE1.

We show one example (Figure 4) and an additional two examples (Figure S10) for each of the CNV association type classifications covering a variety of SNP-CNV correlation patterns and differential signal strengths using locus zoom-style plots but with the focal point being the lead exon from the fine-mapped CNV region. For SNP-CNV-near and SNP-CNV-far classes, we show significant association signals for CNVs and SNPs (Figures 4A and 4B) restricted to a single exon of the *ACAN* gene association to height and a region including *C4A* and *C4B* genes with an association to the FEV/FEC ratio (see Data S3 for a further description).

Next, to investigate which gene-to-trait associations would be detectable by CNV association testing only, we classified novel CNV associations as CNV-allele at the *OCA2/HERC2* locus, which is described in detail above. Interestingly, although there is strong evidence of association, with similar signal strength, from both CNV- and SNP-based tests at *HERC2*, there is very little correlation between the two variant types (Figure 4C), suggesting that these associations are likely to be operating via different functional mutations. For the CNV-only class, we discover a highly specific association to chronotype within the *SPDYE1* gene on chromosome seven where there are no tagging SNPs and no SNPs associations within 1 Mb (Figure 4D). The *SPDYE1* gene has no previous association to measures of sleep patterns and very little description of CNVs at this location; however, a related gene, *SPDYE6*, has been associated with insomnia via SNP GWAS in a much larger sample set (1.3 million samples).^81^ We also discovered significant CNV-only associations at *SPDYE6* and the directly adjacent region containing the *POLR* and *SPDYE2B* genes, which have been previously associated with chronotype by SNP GWAS in the UK Biobank.^82^ Here we provide strong evidence that CNVs at *SPDYE1*-, *SPDYE6*-, and *POLR*-related genes are associated with a measure of chronotype in the UK Biobank and may act in a dose-dependent manner to influence an individual’s sleeping pattern.

We also present two additional examples of each of the different CNV association classes (Figure S10), including SNP-CNV near associations to hair color at the *SPG7* gene^83^ and to alcohol consumption at a 0.8-Mb region including four fine-mapped CNV regions involving the *NPIPB6*, *NPIPB7*, *NPIPB9*, and *SH2B1* genes;^79^^,^^84^, ^85^, ^86^ SNP-CNV-far classifications for hair color at the *TRIM49C* gene and standing height at a fine-mapped region involving the *EVPLL* and *LGALS9C* genes; CNV-allele association types for heel bone density at the *WNT16* gene^87^, ^88^, ^89^, ^90^ and the FEV/FEC ratio at the *HTR4* gene^91^, ^92^, ^93^, ^94^, ^95^; and CNV-only classifications for the FEV/FEC ratio at the *ZDHHC11B* gene^91^ and for standing height at the *CDK11A* gene.

### Competitive SNP-CNV association models

We performed joint CNV-SNP competitive association models for standing height and hair color (see STAR Methods) since these traits include multiple signals genome-wide and have been extensively studied by previous work.^96^^,^^97^ Across 91 exon-level CNV association signals that had at least one significant SNP within 1 Mb around the lead CNV position, we performed eight different models (see STAR Methods). First, we applied a three-component mixture model to the normalized copy number estimates (log2 ratio) to define copy number genotypes (assuming a simple deletion/gain process). Next, we performed joint SNP and CNV competitive models using both the SNP with the highest signal strength against the same trait, in the same samples, and the SNP showing the highest *r*^2^ against the lead CNV exon within 1 Mb.

**Figure 5:**
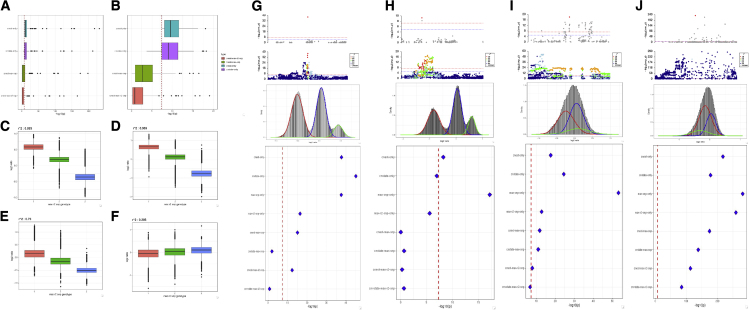
Competitive models for CNV and SNPs using copy number estimates, copy number genotypes, and joint models including SNP genotypes from the most highly correlated SNP or the SNP with the highest association signal for the same trait within 1 Mb (A) Minus log10 p values for four different models: CNest only, copy number estimates only; cnstate only, copy number genotypes (three-component mixture model) only; CNest-max-snp, joint model with copy number estimates and the SNP with the highest association signal for the same trait within 1 Mb; CNest-max-r2-snp, joint model with copy number estimates and the most highly correlated SNP within 1 Mb. (B) Zoomed in view of (A) restricting the x axis to a maximum −log10 p value of 20. (C–F) SNP genotypes from the most highly correlated SNP against the copy number estimate (log2 ratio) for four individual exon-level association signals, further details of which are shown in (G)–(J). (G–J) Finer-grain details for joint models of four exon-level copy number association signals; top panel shows the copy number estimate association signal with the lead exon highlighted in red, second panel shows the SNP genotypes association signal from SNP GWAS tests in the same samples and trait colored by the *r*^2^ of SNP genotypes against the lead exon signal from the copy number GWAS (CN-GWAS), the third panel shows the copy number estimate (log2 ratio) of the lead exon association from the CN-GWAS fitted using a three-component mixture model to define copy number genotypes, and the fourth panel shows the −log10 p value from eight different types of association model: cnstate-only, cnstate-only, max-snp-only, max-r2-snp-only, CNest-max-snp, cnstate-max-snp, CNest-max-r2-snp, and cnstate-max-r2-snp.

After the denoising of copy number estimates into a three-component model, the majority of associations (71 out of 91) showed a lowering of signal with 17 out of 91 sites dropping below genome-wide significance (Figures 5 A and 5B). For most sites, the association signal strength was lowered, with a median −log10 p value reduction of 1.7 for the three-state model; however, there were 20 out of 91 sites that showed a marginal improvement in signal strength with a median increase of 0.65, and one site at the *GOLGA6L4* gene showing the relatively large increase of seven when using the CNV state compared with the CNV estimate model. The drop in p values is to be expected due to the selection of the most associated exon (winner’s curse phenomenon), but it shows that there are no large gains to be made by a more categorical model for discovery.

We show the relationship between copy number estimates and SNP genotypes (Figures 5C–5F) as well as a finer-grained view of the difference in association signals when performing eight different competitive models (Figures 5G–5J). These four examples range from highly correlated and well-tagged CNVs (Figures 5C, 5D, 5G, and 5H) to moderately tagged CNV (Figures 5E and 5I), and finally a poorly tagged CNV (Figures 5F and 5J). For competitive SNP and CNV modeling, the simplest hypothesis is that sites that are well tagged and show association to the same trait are likely to be able to control each other’s signal in a pairwise competitive model. Across the 91 sites, including the SNP with the highest signal strength irrespective of its *r*^2^ can fully control 72% (66 out of 91) of CNV associations (Figures 5A and 5B). When switching the SNP to those with the highest *r*^2^ irrespective of association strength, we observe an increased level of control with 80% (73 out of 91) of CNV signals being reduced below genome-wide significance. Indeed, the most frequent situation is that SNPs that can well tag the CNV in aggregate (across all samples) are best able to control the CNV association signals within a competitive model, with only two cases where highly correlated and significant SNPs are unable to fully control the CNV association (Figures 5G and 5I). As expected for the 18 CNV associations that cannot be fully controlled by either SNP type, the majority show very little tagging, with 80% of these sites having a maximum *r*^2^ below 0.6 for all SNPs within 1 Mb. However, there are two cases where the main assumption that highly correlated SNPs can control the CNV association does not hold true (Figures 5G and 5I).

We show four examples of different types of association control from these competitive models. First, a case where neither SNP type included in the models can fully correct the CNV estimate signal, where we assume the SNPs are tagging the CNV (Figure 5G). Next, an example where both SNPs, either the most significant or the most highly correlated, can fully control the CNV association signal and we assume that the CNV tags the more significant SNP (Figure 5H). It is worth noting that the most significant SNP is found in intron 1 of the *DOCK8* gene, whereas the CNV signal and the tagging SNP are both found in the neighboring *CBWD1* gene, and that both genes have been found to be associated with the trait (hair color) by previous SNP GWAS testing.^83^^,^^96^ We also show an example of a CNV association where neither SNP can fully control the CNV association, but the highest-correlated SNP is able to push the copy number state joint model down just below genome-wide significance (Figure 5I). The highest signal SNP is found closest to a different gene upstream of the CNV signal and there are multiple tagging SNPs surrounding the CNV locus, which we assume all tag the CNV. Finally, we show an example of a highly significant CNV association signal that has very little SNP tagging around the locus and is not able to be controlled by either SNP in the competitive joint models, supporting our general assumption that SNPs that cannot well tag nearby CNVs are unlikely to be able to control any CNV association even if both variant classes show significant associations to the same trait (Figure 5J).

Here we have shown results from combined SNP and CNV association testing across close to 100 significant exon-level CNV associations. We have shown that the most obvious assumption that nearby tagging SNPs are often able to control CNV associations holds true but that more complex situations exist where aggregate variant correlations are not sufficient to predict the interactions between them in relation to trait association testing. We have also shown that it is possible to use copy number estimates in a dosage-dependent linear model as a reasonable proxy for the underlying copy number state distribution, and, by applying similar methods to the highly successful SNP GWAS approach, it is possible to discover novel CNV associations, adding additional supporting evidence for SNP-based trait association mapping further delineating the underlying genome architecture and variant interactions for trait associations.

## Discussion

In this paper, we present a robust CNV-to-phenotype discovery process that uses NGS information that is analogous to the traditional SNP-based GWAS. This paper therefore complements the long-standing use of CNV in rare disease discovery^98^ and provides a higher-resolution view of common CNV than established SNP array-based methods.^21^ A key foundation is a robust normalization procedure that can handle the diversity of DNA presentation and extraction states in a large cohort. Armed with this normalized copy number level, we decided to model the complexity of CNVs in the genome as a linear dosage variable; this model is obviously an approximation to the reality of structural variation, but it allows consistency of statistical approach and the same degree of freedom across the genome between loci, and means that similar covariates, methods, and diagnostic procedures, with similar expected null model properties to SNP GWAS, can be used.^99^ Our resulting linear dosage model produces well-calibrated statistics for both quantitative and qualitative traits, where most associations fit the expected null model. The minority of associations where one can confidently reject the null model at a genome-wide significance level include many well-known individual CNV associations, with a total of 862 associations across 78 different human traits.

We have illustrated the large-scale discovery of CNV associations with 12 examples in the main text and an additional 18 examples in the supplemental information. The examples vary from well-established CNV loci (e.g., *LPA* with heart disease loci) through partially understood CNV loci (e.g., the *UGT1A* gene in porphyrin and bilirubin metabolism) to very credible associations to paralogues with the same phenotype (the RCHE gene in pregnancy complications) or credible novel alleles in a gene with robust association to a phenotype (HERC gene with hair color). Across most UK Biobank traits that we tested with sufficient sample size, we obtained strong discovery signals for CNV associations genome-wide, and, importantly, all signals were within exons providing a direct link to genes. Most (82%) of these fine-mapped association regions contained a single gene and sometimes single exons; however, when there are multiple genes across individual regions it can be hard to determine which gene is most responsible for the association. One such case is shown with the association of ICD10 code E80 (disorders of porphyrin and bilirubin metabolism) to multiple *UGT1A* genes. Although these minority of situations can be challenging to interpret, since we achieve exon resolution within our association tests, it is possible to rank individual exons (or genes) based on association signal strength or effect sizes. There are numerous other examples in the supplemental information, and the full information of the discovery processed here is available both via UK Biobank return of results and via the GWAS catalog. Even so, we have chosen only a subset of phenotypes present in the UK Biobank, itself only one cohort; to enable broader discovery by others, we have released CNest as an open-source package and provided portable workflows consistent with GA4GH standards. One of the central components of the association testing reliability is the generation of accurate copy number estimates. CNest uses a dynamic reference approach similar to previous work on microarrays and sequence datasets,^100^, ^101^, ^102^ selecting an optimized set of internal samples to use as baseline copy number measures. We hope to build out more extensive user-friendly tutorials, including the practical and necessary aspects of QC before discovery. We encourage the community to examine the results we have presented here and use the software to make more discoveries.

When comparing our results with other studies into CNV association in the UK Biobank using WES for CNV detection and association testing focused on asthma,^103^ we see good agreement in association signals for many of the asthma-specific associations we have reported in this study. When comparing results with CNV studies using SNP genotyping arrays,^56^^,^^57^ we can confirm 63% of previous CNV associations made across nine quantitative traits. For those associations that were not confirmed by CNest, it is difficult to know whether these are real false-negatives from the exome association testing or are due to other technical or model choices. Some possibilities are that both SNP array-based studies used more than twice the number of samples, as well as differences in the type of signal that can be derived from the two platforms (WES versus SNP) where, for example, there may be certain exonic baits that are challenging for NGS due to differences in capture efficiency.^104^ One other important factor is, unlike the two SNP array studies, we deliberately choose to assume additive effects, allowing us to place our results into a similar framework most often used in SNP GWAS, and did not attempt to model any non-linear effects. Nevertheless, we were able to obtain many more associations and at far higher resolution during this study, providing strong evidence that many copy number variable locations throughout the genome do associate with both continuous and discrete human traits. Further research is needed to fully catalog CNV associations across large cohorts such as the UK Biobank and beyond, and there are also many further interesting methodological questions to address where it may be useful to integrate information across multiple platforms for CNV discovery and association testing.

We have shown that it is possible to bring CNV GWASs into a similar framework as genome-wide SNP tests for trait association mapping in large cohorts, opening several new avenues of investigation into combined modeling of CNV association signals in human traits. We were also able to look at the correlation between SNP and CNV discoveries by performing association testing independently and jointly using the same sample sets and traits. Here it is possible to start to estimate the contribution that both types of variations have on trait associations in a large cohort and to gain some insight into the different types of interactions that can occur. As expected, for the CNV associations with some level of correlation to an SNP, there are complex relationships between SNPs and CNVs, and most CNVs that can be well tagged are relatively common in the population. In some cases, the SNP associations can completely explain the CNV association, whereas, in other less frequent situations, the CNV association cannot be recapitulated by any SNP. This latter case is consistent with multiple CNVs arising on different haplotypes, where the CNV association appropriately aggregates the CNV information in a way that is far harder to achieve via tagging SNPs. Even in the cases where the loci are discoverable by SNP methods, and the SNPs tag the CNV, the large impact of deletion or expansion of an exon makes CNV an interesting potential functional change.

CNV has long been known as an important aspect of germline DNA variation, and has long been a key part of rare genetic disease discovery and diagnosis. The system we have proposed here, CNest, can robustly find associations of CNVs to common phenotypes in large cohorts, but we have only started in providing a full catalog of these results. We encourage the community to explore the discoveries we have made in this paper, to use CNest to make more CNV associations in both UK Biobank and beyond, and to help extend the CNest method further to provide a more comprehensive view of human variation.

### Limitations of the study

In this study, we have limited ourselves to associations consistent with a standard additive model that allowed for a more natural comparison with SNP GWAS results; however, there are known examples of CNV loci where both deletion or duplication (and in particular truncating duplications) can have a negative consequence on human phenotypes.^105^ In further work, it will be interesting to expand the CNest framework to include additional models that can account for non-linear effects (such as U-shaped distributions or “mirror models”), which is very likely to result in an increase in the number of significant associations that can be made. Unlike most CNV GWAS studies to date, we have included association testing across multiple quantitative and binary traits, discovering many new associations across a large range of phenotypic measures. Similar to SNP GWAS,^106^ we were able to discover more associations for quantitative traits compared with disease-related binary codes; however, we did make several discoveries related to human disease code, highlighting that certain genomic regions could contribute to disease progression in a dose-dependent additive manner.

The ability to jointly model SNPs and CNVs in the same framework will more easily allow for integration of these two types of variation. An obvious extension is to polygenic risk scores (PRSs) for traits, where the linear model for CNVs naturally fits with the additive linear SNP loci in a PRS.^107^ However, care needs to be taken over ascertainment and modeling for PRSs, in particular for certain traits such as blood-based cancers where the normalization procedures we have employed for CNV association might not be robust enough to distinguish germline from somatic changes in cancer risk. Importantly, this means care needs to be taken about the time of blood sample compared with the onset of diseases in constructing such PRSs. Another extension will be using these linear variables as instrumental variables in Mendelian randomization techniques to understand causality between physiological processes and often disease outcomes.^108^ A similar concern on normalization techniques needs to be considered, along with careful consideration of the assumptions behind any instrumental analysis.

## STAR★Methods

### Key resources table

**Table:** 

REAGENT or RESOURCE	SOURCE	IDENTIFIER
**Deposited data**		

UK BioBank Whole Exome Sequence (200,000 release)	UK BioBank	Data field: 23143
GWAS Catalogue summary statistics ftp://ftp.ebi.ac.uk/pub/databases/gwas/summary_statistics/GCST90103001-GCST90104000/GCST90103348	GWAS Catalogue (master summary stats file)	Project: GCP000324
UK BioBank Data returns (to be submitted on publication)	UK BioBank	Application: 49978

**Software and algorithms**		

CNest: https://github.com/tf2/CNest	GitHub	3c2b94a
CNest: https://app.terra.bio/#workspaces/ga4gh-cnest-test/cnest-terra	Terra Bio	NA
CNest: https://hub.docker.com/repository/docker/tomas81/cnest	Docker Hub	tomas81/cnest:dev
CNest: https://zenodo.org/record/6770130#.YsLe6-zMJMM	Zenodo	https://doi.org/10.5281/zenodo.6770130
ViteRbi: https://zenodo.org/record/6794409#.YsLliuzMJMM	Zenodo	https://doi.org/10.5281/zenodo.6794409
Examples of association tests and plotting CNest results: https://zenodo.org/record/6806357#.YsavNuzMJMM	Zenodo	https://doi.org/10.5281/zenodo.6806357

### Resource availability

#### Lead contact

Further information and requests for resources should be directed to and will be fulfilled by the lead contact, Tomas Fitzgerald (tomas@ebi.ac.uk).

#### Materials availability

CNV GWAS summary statistics and fine mapped association regions are included in the supplementary material of this paper.

All association results, including all sites tested irrespective of association signal strength, have been submitted to the GWAS catalogue and are available for download under the project ID: GCP000324.

CNV calls and copy number estimates will be made available via the UK BioBank data return and linked to UK Biobank application number: 49978.

### Method details

#### Sample cohort and phenotypes

For this study, we used 200,624 Whole Exome Sequencing datasets from the UK Biobank 200k release generated using the IDT xGen Exome Research Panel v1.0 including supplemental probes and sequenced with dual-indexed 75 × 75 bp paired-end reads on the Illumina NovaSeq 6000 platform using S2 and S4 flow cells^109^. We used the aligned CRAM files from the OQFE pipeline which aligned and duplicate-marked all raw sequencing data (FASTQs) against the full GRCh38 reference in an alt-aware manner as described in the original FE manuscript^110^. These aligned sequence datasets were used as the primary input in the CNest pipeline (**details below**) for exome-wide copy number estimation and CNV calling. Phenotypes were extracted and linked to the copy number data under UK Biobank application number 49978, resulting in a total of 78 different traits (24 quantitative and 54 binary) that we tested for CNV association (Tables S2).

#### Genetic data processing and copy number estimation

We used CNest (full source code available: https://github.com/tf2/CNest.git) to carry out large scale copy number estimation in the UK Biobank 200k WES release. This program was designed to provide accurate copy number estimation from very large NGS (WES and WGS) datasets. The first required step is to extract read coverage information for all genomic locations of interest, to do this CNest makes use of the samtools and htslib libraries^111^ implementing a custom coverage extraction method that importantly filters reads based on several samtools alignment flags. The main flags of interest are BAM_FPROPER_PAIR, BAM_FDUP and BAM_FSECONDARY where we ensure that aligned reads have a MAPQ greater than 1, are primary alignments with proper pairs and are not PCR duplicates.

After extraction of coverage information, the first important step is to classify the sex of each sample based on the relative coverage on chromosome X. Here CNest implements a simple k-means clustering for the initial classification and quality control steps. This initial step results in the classification of two states relating to 2 or more and 1 or less copies of chromosome X (although less than 1 copy of chromosome X is biologically incompatible there can be data quality issues to account for when processing large volumes of data). CNest also implements a prototype automated classification to detect sex chromosome auniopoly which is based on the ‘abberant’ cluster^112^ however we highly recommend that the sex classification in checked by a human before moving onto the next steps. This is because all datasets are different and will contain a variety of sex classification types (Figure 1A), as standard across large numbers of samples we observe 3 types of sex chromosome dosage exclusion types and classify samples into either male or female, or ambiguous low, ambiguous mid, ambiguous high. All samples that are not classified as either male or female are removed from the subsequent steps.

The next step is to derive sample specific dynamic reference datasets, briefly, CNest uses an optimisation process to select groups of appropriate datasets to make up individual references (or baseline) estimates across the genome for each sample individually, similar approaches have been proposed and successfully applied to derive copy number estimates from coverage level data. One such approach can be found within the virtual reference genome (VRG) approach from the C-SCORE method^100^. It is worth noting that by using this type of approach it is impossible to directly obtain the true copy number at any genome location, rather it allows the optimisation of relative copy number estimates that are often (particularly if the reference set size is sufficiently large) likely to be a good reflection of the underlying copy number state distribution.

During its baseline reference selection processes CNest estimates the overall correlation of coverage information between all samples, applies a wavelet model for estimating the scale of genomic waves, and implements a dose response optimisation using sex mismatched samples and the expected single copy dosage change. This process has been designed to be extremely efficient across very large numbers of samples and results in a ranked list of which samples are most correlated in terms of certain coverage patterns and noise characteristics which are assumed to be the ideal set of samples to generate the baseline estimate. The only parameter needed to be decided on at this stage is the total number of datasets to use to generate the dynamic references, for the UK Biobank 200K release we elected to use 2,000 samples within each of the ∼200K dynamic reference datasets. Although it may, in some cases, be preferable to allow the dynamic reference sets to be made up of different numbers of individual samples (which is possible using CNest) we decided to fix this number across all datasets as we wanted to minimise any potential biases within the resulting copy number estimates that could be introduced due to using differentially sized reference datasets.

Following the dynamic reference selection process, the median coverage for all genomic locations across all relevant reference datasets is calculated for each sample individually for matched, mismatched, and mixed sex classifications. These data values are stored in the custom CNest binary format to allow fast random access across the genome and across different sample sets. Finally, the coverage information and reference estimates are transformed into the log2 ratio space and median normalised using the median log2 ratio excluding sex chromosomes for sex matched, mismatched, and mixed estimates. These estimates are again saved back into the CNest binary file format to allow efficient extraction during the next steps of CNV detection and CNV GWAS testing.

For CNV calling CNest implements a single custom designed 3 state Hidden Markov Model (HMM) to call losses and gains, the basic implementation of which can be found (https://github.com/tf2/ViteRbi). In our hands this HMM model has been highly reliable across a number of different detection applications, and it is extremely efficient in terms of speed and memory usage. We apply a few important steps during the HMM training (using the EM algorithm) to further improve the reliability of the model. Primarily these include training the model independently for each sample by using all the log2 ratio estimates of all samples that made up the dynamic reference for that particular sample. By doing this we aim to increase the accuracy of the transition probabilities by giving the EM algorithm sight of large numbers of highly similar datasets. Importantly the log2 ratio estimates that we use during this training phase are those generated using the sex mismatched dynamic reference, ensuring that there is always at least one large single copy number loss/gain event present in all training datasets. Having trained the HMM for each sample independently using this approach we apply the forward backward Viterbi algorithm to call the most likely sequence of state paths across each sample independently. The result of this process is the state calls (0,1,2) for every genomic position of interest across all samples, we then apply some merging criteria to obtain both state classification and CNV regions (CNVR) across the genome. In fact, although CNV callers will often impose some complex merging criteria to account for outlier points within each called CNVR (e.g. by allowing a certain number of outlier copy number estimates within a CNVR) we are so confident in the performance of the HMM that we simply merge consecutive state calls without any additional complex merging rules. As illustrated in Figure S4, this process does not result in over fragmentation of CNV regions.

#### CNV merging, frequency estimation and copy number principal component analysis

Having obtained reliable copy number estimates and CNV calls across all samples we apply some cross sample merging criteria to allow us to generate merged copy number events (CNVEs) with frequency information attached. To do this we merge losses and gains separately across all samples using an iterative 50% reciprocal overlap rule, building up sets of CNVRs across all samples where all member segments (calls) within each set must share at least 50% of its boundaries (start and end positions) with at least one other segment within the set. Once we obtain full closure of the set, when no additional segment can be added to the set, we adjust the final start and end position by 80% of the inner to outer start and end positions. Finally, we calculate loss, gain and overall CNV frequency and standard errors for each CNVE resulting in a set of CNV regions across the genome with frequency estimates that can be used in some of the subsequent analyses (e.g. PCA and Association testing).

On top of the frequency estimate for all merged CNVEs we also assign frequency measures to individual bait regions where we can calculate the frequency that each bait is included within any copy number event. This gives us two different sets of CNV type and frequency datasets that can be used to perform principal component analysis (PCA) across the sample space. PCA is often used in SNP GWAS to control for population structure and other technical (or sample level) variation that is less well understood but that is important to control for during genome wide association scans. Similarly, it is important to correct for larger scale differences between copy number estimates across large datasets for CNV GWAS analysis. Often during SNP based PCA the sites get filtered or subsampled to allow efficient PCA to be performed, for CNV analysis it seems important that we can run PCA analysis using both commonly variable CNV regions and rare regions separately. This is due to an observation that when including all CNV sites in PCA, the commonly variable positions tend to suck out a lot of variation and get overrepresented in the first PCs. We used iterative PCA to perform several different types of PCA using both bait level and CNV call level copy number information stratified by frequency estimates. Overall, we find that PCA based on commonly variable positions are better able to capture sample level information such as population structure, whereas PCA based on rare regions can account for cryptic sample differences which are likely due to certain noise properties of the data that we were unable to accurately model during the previous steps.

### Quantification and statistical analysis

#### Genetic association testing

One major point of the CNest methods and approach is that by working with copy numbers in this way we have been able to employ genetic association testing methods like the SNP based GWAS methods that have been applied with great success over many years. For CNV we can use several different estimate types to perform large scale genome wide association tests. Although it would be possible to develop methods for copy number genotyping (i.e. actual copy number states) due to the way we have set up our large scale approach we are not able to accurately determine the real copy number of any individual genome position. Rather we have well calibrated relative copy number estimates across large numbers of samples that can be used to search for associations against any given traits.

We set our models up in a few different ways but always (in this study) by using standard linear and logistic regression techniques, although this choice is potentially suboptimal (Discussion) this was done deliberately to ensure that any CNV association signals follow the general additive model (where the copy number estimate must display a linear relationship against the given trait). All models were applied to unrelated samples from the PCA-defined European cluster (SNP PCs 1 and 2). For quantitative traits we use generalised linear models with covariates and use both the bait level copy number estimate (log2 ratio) and the copy number estimate (mean log2 ratio) for all merged CNVEs across all samples as the test variables. The standard set of covariates we include are sex, age, sequencing batch, the first 10 PC from SNP based PCA and the first 10 PCs from CNV PCA for both rare and common sites separately. Additionally, to ensure that outliers in the phenotype distribution do not impact our association tests, for all quantitative traits we apply an inverse rank normalisation.

For SNP based association tests in exactly the same sample sets we used bgenie^113^ for quantitative and regenie for binary case control trait tests^106^. Imputed SNP genotypes from the 500K UK Biobank release^113^ were remapped to genome build hg38 using the UCSC liftover tool^114^ prior to sample selection based on the 162,633 samples that were used in the CNV association tests. In both cases, for quantitative and qualitative tests, we followed the standard SNP filtering recommendations, including only bi-alleilc SNPs with a minor allele frequency greater than 1%. Association tests were run across the main traits of interest and the genome wide significance cut-off of 5e-08 was used to define associations between SNPs and traits.

#### Definition of the association significance threshold

To justify the use of the widely accepted genome wide significance threshold of 5e-08 for significance testing in this work we looked at how our results would change when using three different p value correction approaches. We assessed the use of a stringent Bonferroni correction, the Benjamini Hochberg (BH) false discovery rate (FDR) based approach and permutation tests.

Overall, applying the stringent Bonferroni correction only slightly lowered the significance threshold to a value of 3.35e-08 and had very little effect on the number of significant exon level associations for most tests (Figure S11) with a median decrease of 0 (mean decrease of 1.19 and maximum decrease of 21) across all traits. However, applying this stringent correction did result in the exclusion of 0.5% (45/862) fine mapped regions across 18 of the 78 traits tested, after correction only 0.5% (4/78) of those traits had zero remaining significant associations at the specific loci since each of these 4 traits only had a single low level exon signal. Next, we applied a 0.01 FDR based BH correction to each association result independently, again we observed highly consistent results for most traits with a median increase of 1 significant exon signal across all traits (Figure S11B). For the majority of traits, the BH correction resulted in less stringent thresholds than both the genome wide and Bonferroni approaches (Figure S11C), and in most cases resulted in either the same (28 traits) or increased (40 traits) numbers of exon level signals that would be defined as significant. For some traits (8/78) the number of additional significant associations for the BH correction was substantially higher (greater than 100 additional exonic signals) suggesting that there could be some value in applying an FDR based correction. Finally, we performed 100 rounds of permutations on 4 main traits (hair colour, height, MI and Asthma) where we randomly ordered the phenotype measurements or case labels and ran our standard linear or logistic regressions models across 100 different random sets for all 4 traits. In all cases after 100 rounds of permutations there were zero signals that passed a genome wide threshold of 5e-08 (Figure S12) indicating that this value is suitable for use in the definition of copy number associations.

Although it may be possible to use a less stringent threshold (such as BH or permutation based) and to obtain a greater number of copy number based trait association, we preferred to remain highly stringent. Copy number associations often display a similar association pattern to SNP GWAS tests genome wide where close by exon signals are highly correlated and associate to the same trait (LD peaks) and the use of the 5e-08 significance value had the additional benefit of allowing us to apply the same definition of significance for both SNP and CNV based association tests.

#### Identifying associated genetic loci and fine mapping

We have developed a set of tools that build on top of the CNest framework to allow large-scale genome wide association testing for CNVs - CNwas. These tools perform several of the important steps described above - namely CNV merge, PCA and GWAS testing using regression models. Since we have placed CNV GWAS analysis into a similar framework to that often used for SNP GWAS analysis we can make use of standard approaches for genetic loci detection and quality control. Firstly, we use the accepted genome wide significance threshold of 5e-08 to define associations between copy number and traits, although in this case we could theoretically lower this cut-off by using, for example, an FDR or permutation approach we preferred to remain highly stringent for the results we describe in this work (see definition of significance threshold). It also now becomes possible to use standard diagnostic approaches to association results, such as QQ plots and permutation. We apply these standard approaches to the CNV association results described here and see that in general the distributions of p values from our association tests are well controlled. For some tests we do see a degree of inflation and calculate the inflation factor - lambda - for all tests (Table S2), overall for the majority of tests we get inflation factors below 1.13 which is generally considered to be acceptable in GWAS tests^115^ and for cases where the inflation factor is above 1.13 we suggest that a level of caution is used when interpreting these results.

We did not perform fine mapping of SNP based association signals as it is not a focus of this work to provide SNP based GWAS results, however we did fine map CNV signals to define fine mapped regions of CNV association that we report (**GWAS catalogue**) and that we could use for investigation into CNV compared to SNP level signals during the next steps. Because our genomic test loci are, by definition, in coding regions of the genome we choose to use a relatively simple approach for fine mapping CNV associations in a gene/exon centric way. First, we merged all directly adjacent significant signals that had no intervening signal below the significance threshold, next we merged all significant signals that were found in the same gene(s). This resulted in a fine mapped list of significant copy number regions that can contain a single exon, multiple exons within a gene or multiple exons across multiple genes, to be clear these regions do not always contain the full coding region of the gene however any intervening not significant signals between two significant signals within the same gene are merged as that intervening region is assumed to be important for the underlying CNVs. For reporting purposes, the -log10 p value is reported for the lead exon signal within each fine mapped region and tests for correlation between SNP and CNV signals in the next section are always performed in relation to the lead CNV signal for each fine mapped region.

#### Comparison between SNP and CNV association signals

One question that we wanted to address with this work is that of how SNP and CNV associations for the same traits interact with each other and how many of the CNV specific association results would have been detectable using standard SNP GWAS tests. To explore this we defined a set of classification rules to allow us to classify each fine mapped CNV region as either, CNV-only (not detectable using SNP GWAS), CNV-alelle (detectable signal from SNP-GWAS but very hard to discover using tagging variation), SNP-CNV-near (detectable by SNP GWAS and very likely to be fine mapped to the same gene), SNP-CNV-far (detectable by SNP GWAS however likely to be mapped to a different gene).

First, we calculated r^∧^2 between the lead CNV signal and all SNP genotypes within 1 MB. For the CNV-only type, if no significant SNP signal within 1MB was closest to the gene from the lead CNV signal, irrespective of the r^∧^2 value, the fine-mapped region was classified as CNV-only i.e. not detectable by standard SNP GWAS. Regions are classified as CNV-allele if there were significant SNP associations closest to the gene but if none of those SNP had an r^∧^2 greater than 0.6 i.e. association is detectable by both SNP and CNV GWAS but are not tagging. Next, if any significant SNP within 1MB did have an r^∧^2 greater than 0.6 and if any of those SNPs were closest to the gene(s) inside the fine mapped CNV region than the region was classified as SNP-CNV-near i.e. the association signal was taggable by a SNP that associated with the same trait and was highly likely to be assigned to the same gene. Finally, if there were significant SNPs within 1MB that have an r^∧^2 greater than 0.6 but if all those SNPs were closer to a gene that was not within the fine-mapped CNV region then we classify these as SNP-CNV-far. It is worth noting that we set our SNP to CNV r^∧^2 cut-off quite low at a value of 0.6, this is because genome wide we observe relatively low r^∧^2 between SNPs and CNVs and although there are numerous cases of very well tagged CNVs (r^∧^2 > 0.9) for these results we decided to be strict with the definition of novel CNV associations meaning that when we classify an associated fine-mapped CNV region as CNV-only we can be very confident that it is not well tagged by any associating SNP within 1 MB and this results in an overall decrease in the number of CNV associations that we classify as CNV-only.

#### Competitive SNP-CNV association models

To look in greater detail at the relationship between SNP and CNV association interactions we performed some joint modelling of two different copy number estimates, log2 ratios and approximated copy number state distributions, by including either the SNP genotypes from the most significant SNP association or the best tagging SNP (highest r^∧^2 values to the lead CNV association) within 1MB around the CNV association location as covariates within a standard linear model. First we selected 91 single exon associations across 2 traits (hair colour and standing height) and extracted the log2 ratio values across all UK Biobank samples included in the association testing and fitted a 3 component mixture model to define the approximate copy number states boundaries, the decision to use a 3 component was twofold, firstly it was to avoid complications in the definition of the number of actual copy number states observed across the 91 sites that would be highly likely to cause problems during mixture model fits and secondly it was to place the copy number state models into a similar categorical distribution to the SNP genotype models. Each sample was assigned a copy number state based on its most likely component from the mixture model and additionally we did not allow any sample to cross the mean of any adjacent component resulting in a 3-state copy number model relating to low, medium, and high copy numbers. Next, we fitted 8 different types of models across all 91 sites where we tested all variant classes independently and additionally included both SNP types (most significant association and best tagging SNP within 1MB around the CNV association) in pairwise competitive linear models for the copy number estimate and copy number state distributions. We extracted both p values and beta effect sizes from each variant type from all the eight different models and carried out a comparison of signal strength across the different models, allowing us to look in more detail at the relationship and interactions between SNP and CNV associations using close to one hundred CNV association discoveries.

#### Comparison to previous CNV association studies using SNP genotyping arrays

We obtained all reported CNV association signals from two previous studies^56^^,^^57^ that were performed using CNVs detected in the full UKK SNP genotyping cohort. First, we remapped these association loci to the latest genome build (GCRh38) using the UCSC liftover tool^116^ with most regions being successfully lifted over (258 / 269 for *Hujoei* et al.^57^ and 114/132 for *Auwerx* et al.^56^). Next, we selected all associations that were made against a trait that overlapped any traits from the CNest results, both studies only tested quantitative traits and we were able to find 9 overlapping traits resulting in 57 CNV associations that we could compare. With these remapped positions and traits, we then interrogated all CNest association signals from the same trait across each region independently and defined the regions as being genome-wide significant if any signal passed the 5e-08 threshold and suggestive if any signal passed the 1e-05 threshold. Although in some cases it may be possible to redefine the critical region within these association loci due to the higher resolution of the exome data, we did not attempt to resolve any region since we did not have access to the raw copy number SNP signals underlying each associated region. Additionally, we performed one hundred rounds of permutation on each of the association regions separately, randomly selecting a region of the genome with the same size and counted the number of times any signal passed either the genomewide or suggestive threshold showing that these regions are strongly enriched for suggestive signals (Table S11).

## Data Availability

The main CNest code base and docker setup can be found here: https://github.com/tf2/CNest This repository contains all the source code and a docker setup as well as a link to a NextFlow workflow. There is a WDL workflow featured inside the Terra platform along with example datasets and a tutorial for getting CNest up and running across a diverse set of computational infrastructure including cloud based systems (https://app.terra.bio/#workspaces/ga4gh-cnest-test/cnest-terra). This tutorial and the additional workflow implementations is linked to from the CNest main repository. All code has been deposited at Zenodo and can be found at the following links, https://zenodo.org/record/6770130#.YsLe6-zMJMM, https://zenodo.org/record/6794409#.YsLliuzMJMM and https://zenodo.org/record/6806357#.YsavNuzMJMM.

## Supplemental information

Document S1. Figures S1–S12 and Data S1–S3

Table S1. Comparison between sex classification and sex chromosome aneuploidy annotations, relating to Figure 1

Table S2. The total number of significant exon signals for all 79 traits and the associated genomic inflation measure, relating to Figure 2

Table S3. The total number of significant exon signals for all tested ICD10 disease codes and the associated genomic inflation measure, relating to Figure 3

Table S4. Fine-mapped association regions for significant associations for hair color, relating to Figure 2

Table S5. Fine-mapped association regions for significant associations for standing height, relating to Figure 2

Table S6. Fine-mapped association regions for significant associations for asthma, relating to Figure 2

Table S7. Fine-mapped association regions for significant associations for myocardial infarction, relating to Figure 2

Table S8. All fine-mapped significant association regions for all UK Biobank main traits tested, relating to Figure 2

Table S9. All fine-mapped significant association regions for all UK Biobank ICD10 disease codes tested, relating to Figure 3

Table S10. Comparison between CNV associations at 57 regions discovered in two different SNP-based CNV association studies, relating to STAR Methods (comparison with previous association studies)

Table S11. Permutation results for 100 permutation rounds for all 57 association signals between SNP-based CNV GWAS studies and CNest, relating to STAR Methods (comparison with previous association studies)

Table S12. Fine-mapped association regions for the six quantitative traits used to assess the relationship between CNV and SNP GWAS testing, relating to Figure 4

Document S2. Article plus supplemental information
