# Supplementary material for: CNest: A novel copy number association discovery method uncovers 862 new associations from 200,629 whole-exome sequence datasets in the UK Biobank
Source: Cell Genom. 2022 Aug 10;2(8):100167. doi: 10.1016/j.xgen.2022.100167 (PMC9903682; doi:10.1016/j.xgen.2022.100167)
Supplement: Document S1. Figures S1–S12 and Data S1–S3 [file mmc1.pdf]

Cell Genomics, Volume *2*

Supplemental information

CNest: A novel copy number association discovery method uncovers 862 new associations from 200,629 whole-exome sequence datasets in the UK Biobank

Tomas Fitzgerald and Ewan Birney

Supplementary Material

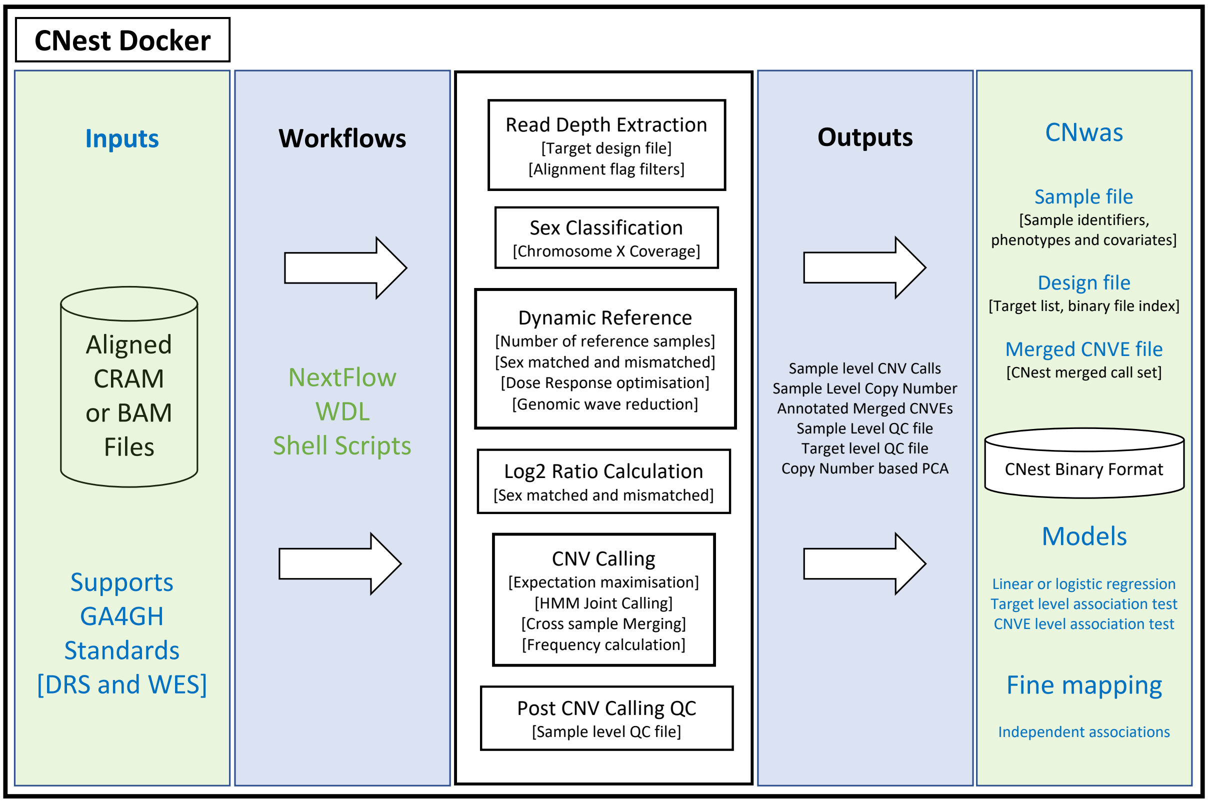

Supplementary Figure 1: *CNest flow diagram showing the main steps performed by CNest for CNV detection and association testing using next generation sequence data – relating to STAR methods (CNest methods). All processes are contained inside the CNest docker and available as workflows in several workflow languages (WDL, Nextflow and shell scripts). The primary input into CNest are aligned sequence files in CRAM or BAM format (and these associated index files). Th workflows run in a number of different ways, where, for example, the WDL workflow runs from start to finish in a single end to end process. Starting from aligned CRAM or BAM files and a target index file specifying the genomic regions to estimate copy number at, CNest via its WDL workflow will run all the individual steps required resulting in, sex classification, sample level CNV calls, merged and annotated CNV events, sample and target level QC files and CNV based PCA results. Next, for association testing via CNwas, a few important decisions need to be made relating to the level of sample and target QC to apply and the covariates to include within the association testing framework (these can be specific to the cohort although we provide some recommended default values). To perform the association testing CNwas needs a few inputs, primarily the output from CNest, copy number estimate in a custom, highly efficient binary format, a sample file including sample identifiers, phenotypes to test and covariates, the target ("bait") design file and the merged CNVEs from CNest. CNwas operates under the same paradigm as tools such as "plink" or "begenie" using 3 primary input file types relating to sample, design and genotype information (".fam", ".bim" and ".bed" style formats). The type of test to perform (linear or logistic) needs to be specified and the process can be split into multiple jobs by specifying a chunking parameter. The result will be target level and CNVE level association results across all traits included in the sample file, which can then be fine mapped using a secondary process.*

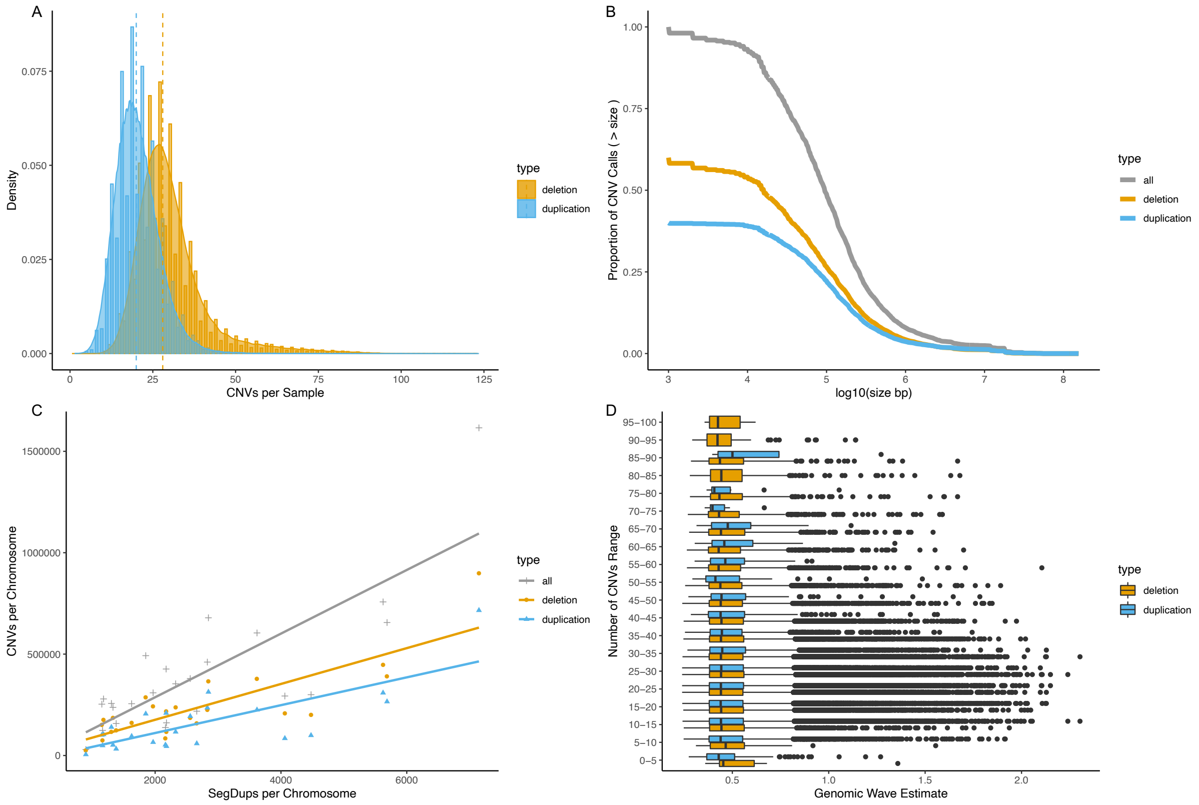

Supplementary Figure 2: *CNV call summary information across ~200K UK Biobank Whole Exome sequences – relating to Figure 1. A: The distribution of the number of CNVs called (deletions in orange and duplications in blue) per sample. B: The log10 of the number of base pairs per CNV call against the total proportion of CNV calls (all calls in grey, deletions in orange and duplications in blue) greater than that size. C: The number of Segmental Duplications per chromosome (GRCh38) against the total number of CNV calls per chromosome (all calls in grey, deletions in orange and duplications in blue). D: The distribution of a genomic wave estimate (IQR of a running median across sample level log2 ratio distributions, using a span of 401 data points) separated across the range of the number of CNV call made per sample between zero and 100 in intervals of 5 CNV calls (deletions in orange and duplications in blue).*

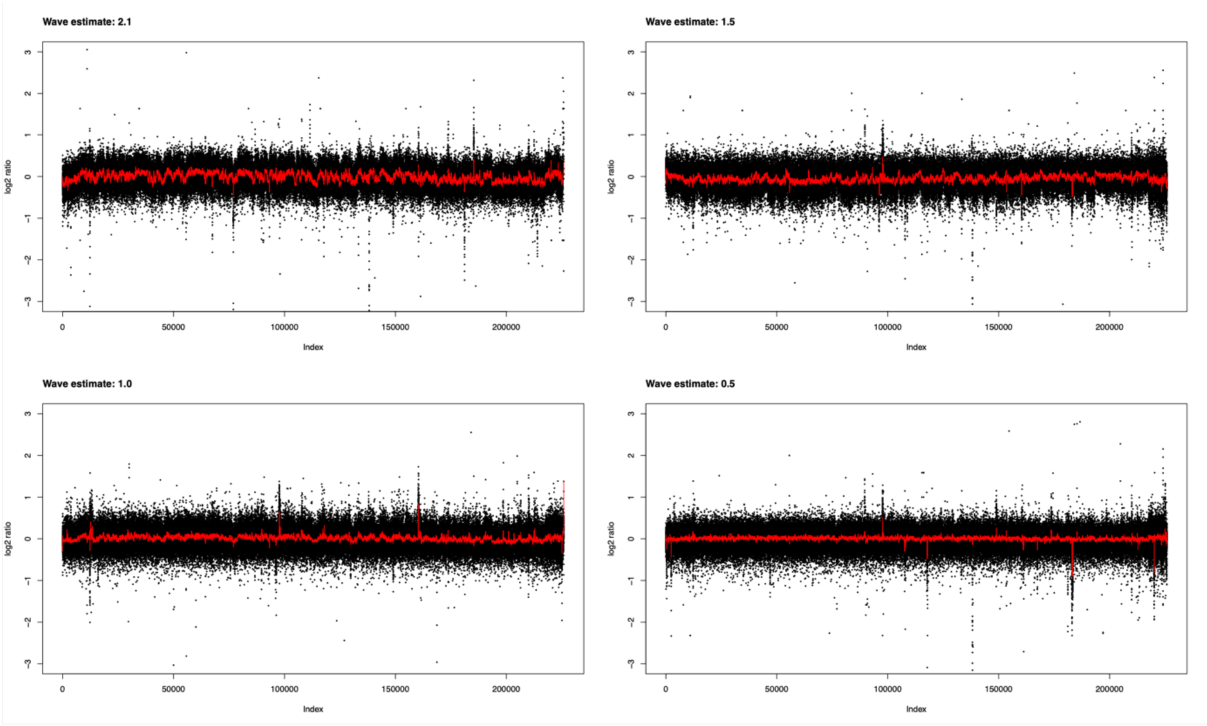

Supplementary Figure 3: *Examples of the log2 ratio values for 4 different samples across the range of genomic wave estimates based on the interquartile range (IQR) of a running median using a 401 data point span scaled by a scaling factor – relating to Figure 1. Upper left: An example of extreme wave characteristics with a wave estimate of 2.1. Upper right: Example of a moderate wave level with a wave estimate of 1.5. Lower left: Example of a mild wave level with a wave estimate of 1.0. Lower right: Example of a sample showing very low level of wave characteristic with a wave estimate of 0.5 Note: wave estimates of 0.5 is where the majority of the CNest normalised sample level log2 ratio copy number estimate in the 200K UK Biobank Whole exome sequence are centred around (**see Supplementary Figure S2D, CNV call summary information, CNV calls**).*

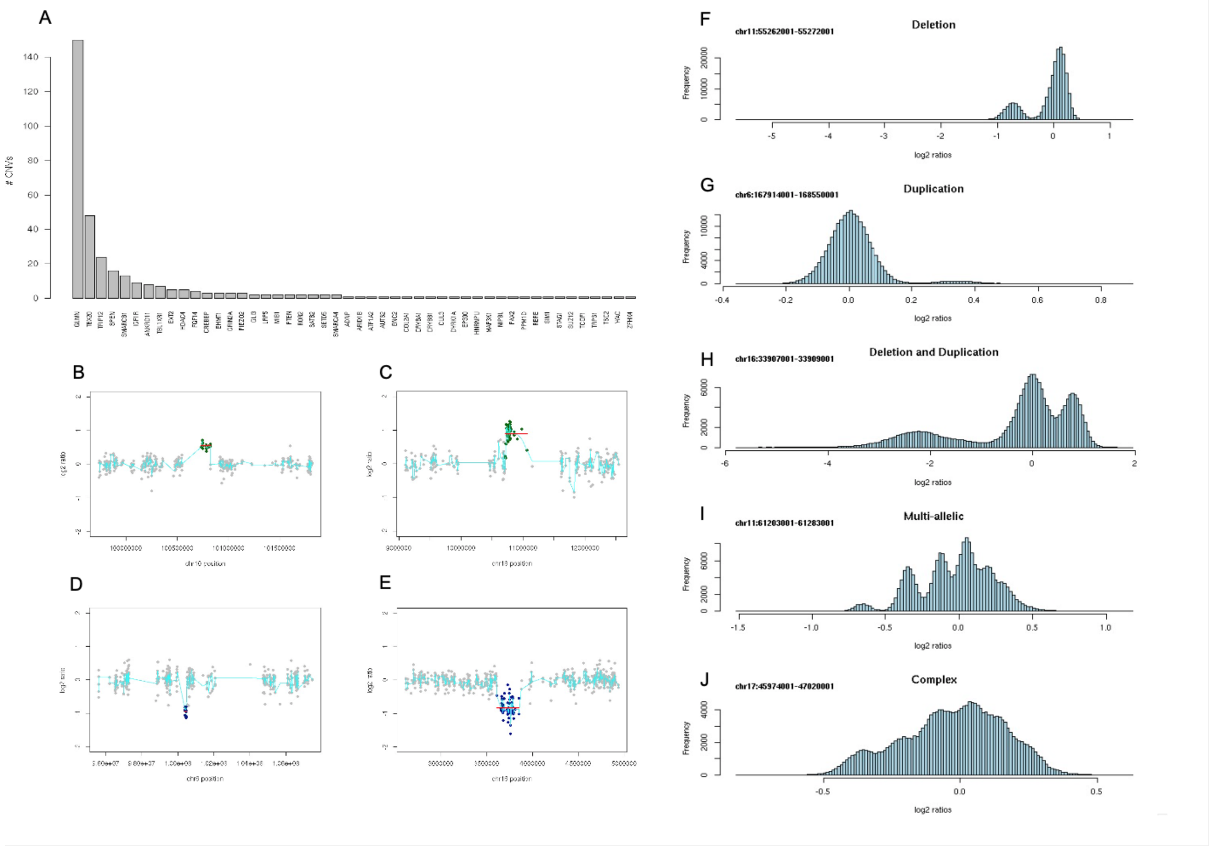

Supplementary Figure 4: *Individual CNV calls and copy number variable locations in the UK Biobank – relating to Figure 1. A: Barplot showing the number of CNV calls overlapping any of 218 monoallelic loss of function genes from the DDG2P (dd gene to phenotype). B: Truncating duplication at the PAX2 gene. C: Truncating duplication at the PIEZO2 gene. D: Deletion at the SIM1 gene. E: Deletion at the CREBBP gene. F: Deletion locus at 11q12.1. G: Duplication locus at 6q27. H: Deletion / duplication locus at 16p11.2. I: Multi-allelic locus at 11q12.2. J: Complex locus at 17q21.31.*

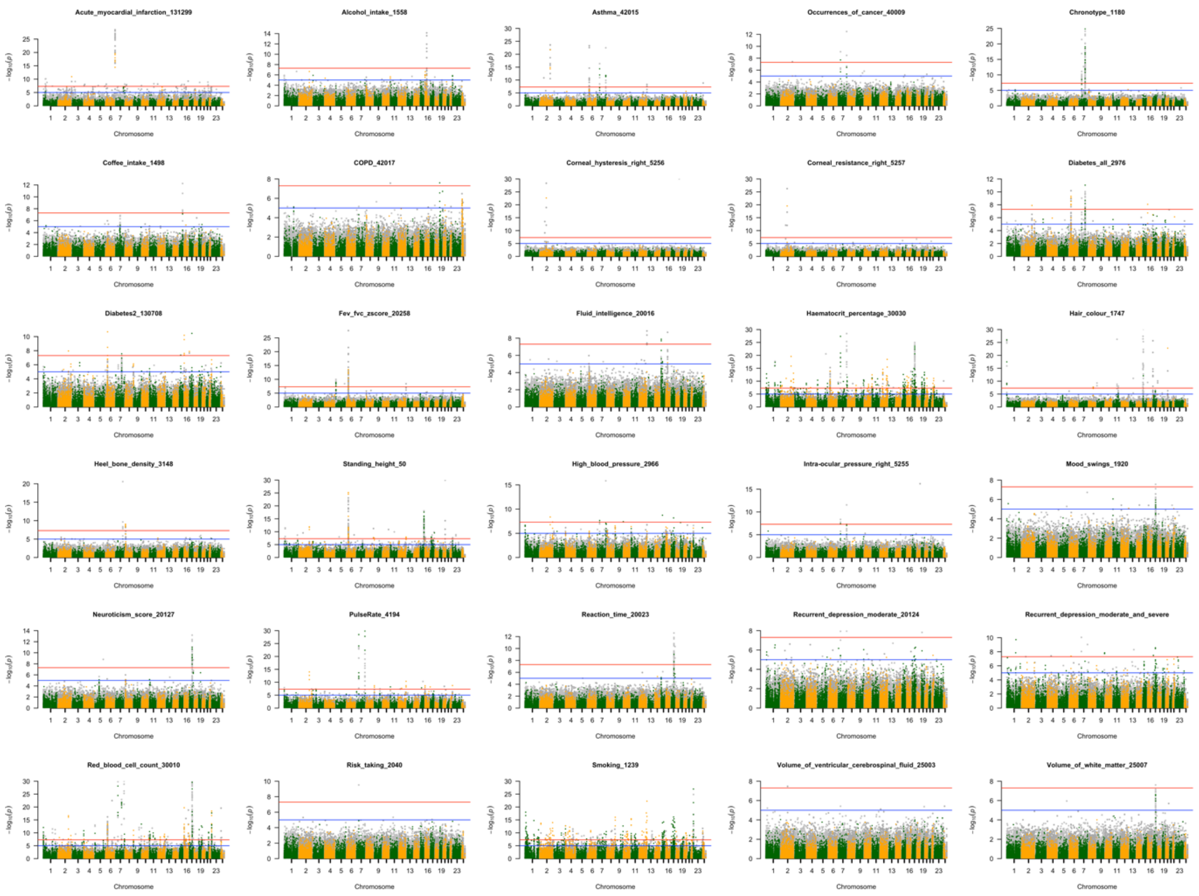

Supplementary Figure 5: *Individual manhattan plots for 30 of the 34 main traits for CNV association in the UK Biobank 200K whole exomes – relating to Figure 2. All plots are pinned to a maximum -log10 p-value of less than 30, meaning that all stronger association signals are not shown but this significantly aids the visualisation across all traits. We exclude 4 traits, showing only right eyes for eye related traits and only red blood cell counts for red blood cell related traits. All the 30 panels have a title showing the trait and all include both p-values from exon level (copy number estimates) trait association testing (grey) and CNV call ("genotype") association testing (green and orange).*

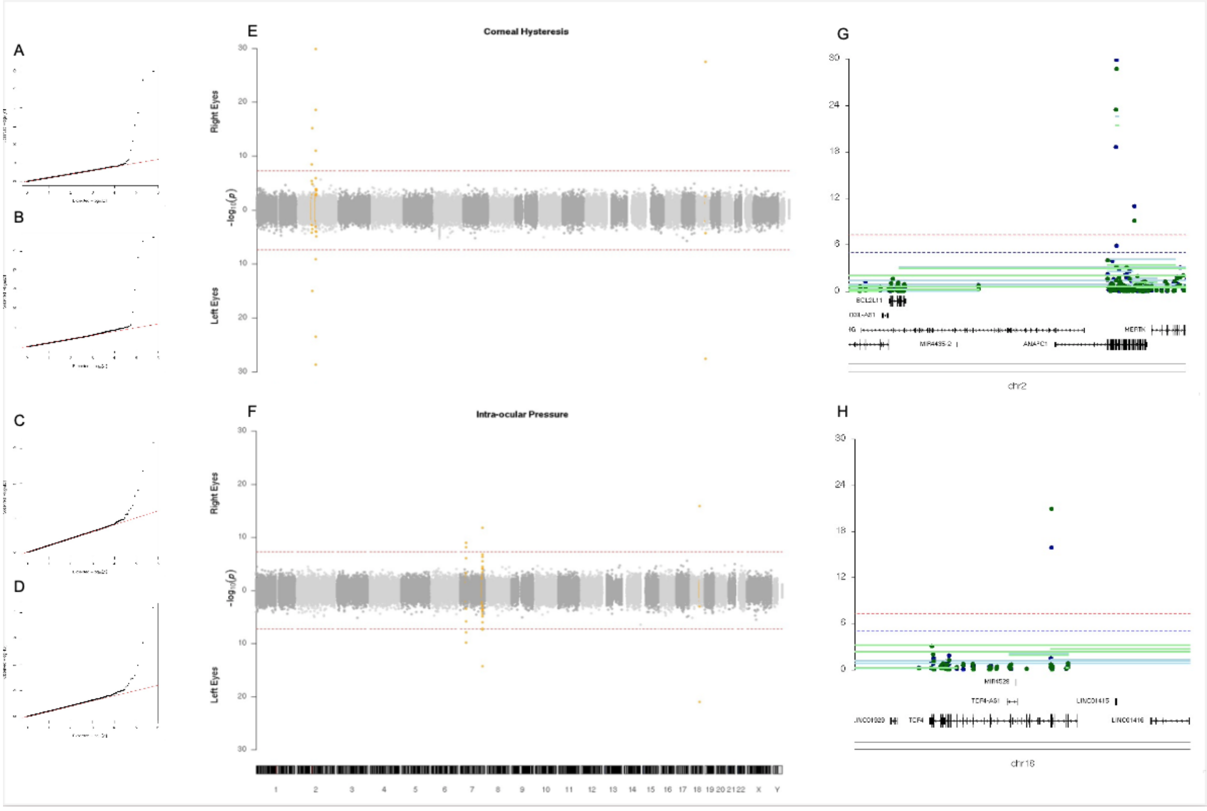

Supplementary Figure 6: *CNV association results for the eye related traits, Corneal hysteresis and Intra-ocular pressure for left and right eyes separately – relating to Figure 2. A: QQ plot for Corneal hysteresis in right eyes. B: QQ plot for Corneal hysteresis in left eyes. C: QQ plot for Intra-ocular pressure in right eyes. D: QQ plot for Intra-ocular pressure in left eyes. E: Bidirectional manhattan plot for Corneal hysteresis in right (top) and left (bottom) eyes. F: Bidirectional manhattan plot for Intra-ocular pressure in right (top) and left (bottom) eyes. G: Locus zoom plot of right eye Corneal hysteresis at the ANAPC1 gene. H: Locus zoom plot of right eye Intra-ocular pressure at the TCF4 gene.*

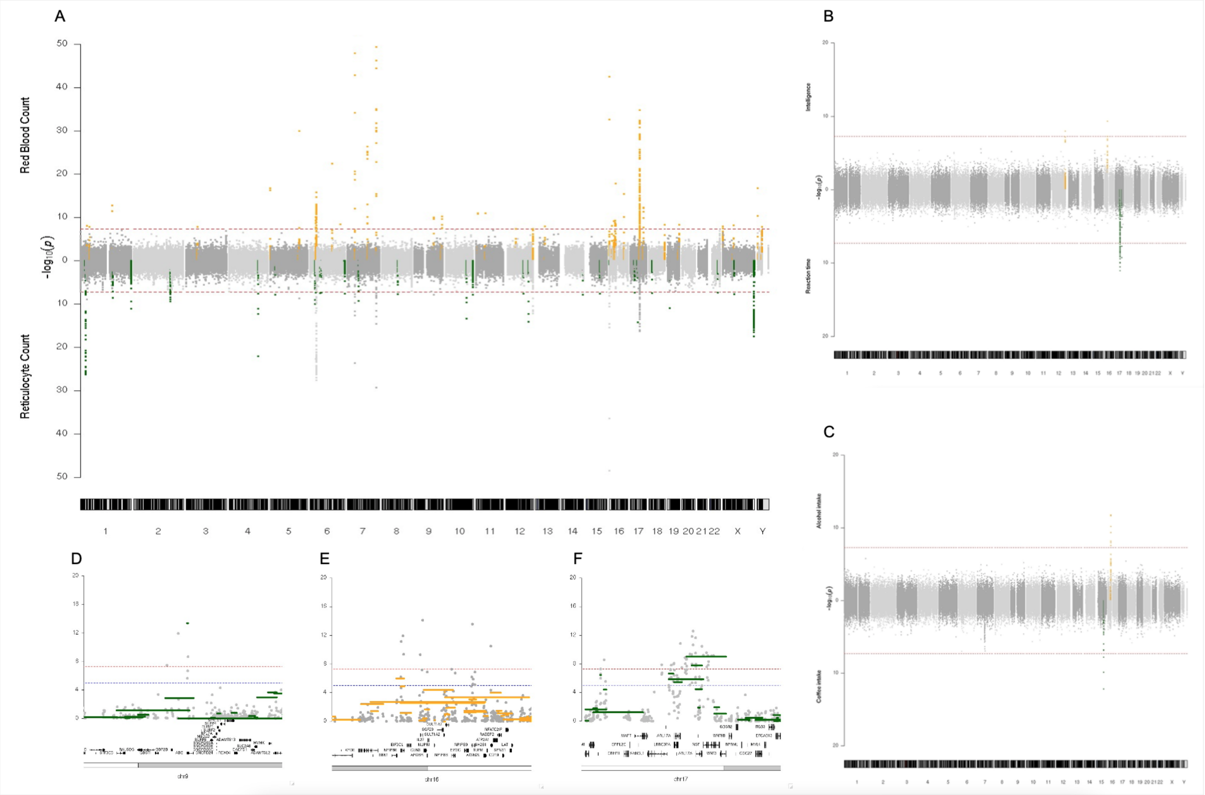

Supplementary Figure 7: *CNV association results for the red blood cell, neurological and behavioural related traits – relating to Figure 2. A: Bidirectional manhattan plots for red blood cell count (top) and reticulocyte count (bottom), fine mapped regions are highlight in orange (red blood cell count) and green (reticulocyte count) with fine mapped regions for reticulocyte count that were also discovered for red blood cell counts not being highlighted. B: Bidirectional manhattan plots for fluid intelligence (top) and reaction time (bottom), fine mapped regions are highlighted in orange and green respectively. C: Bidirectional manhattan plots for alcohol (top) and coffee (bottom) intake, fine mapped regions are highlighted in orange and green respectively. D: Locus zoom plot for red blood cell count at the ABO gene. E: Locus zoom plot for alcohol intake around the NPIPB6 gene. F: Locus zoom plot for reaction time around the ARL17B gene.*

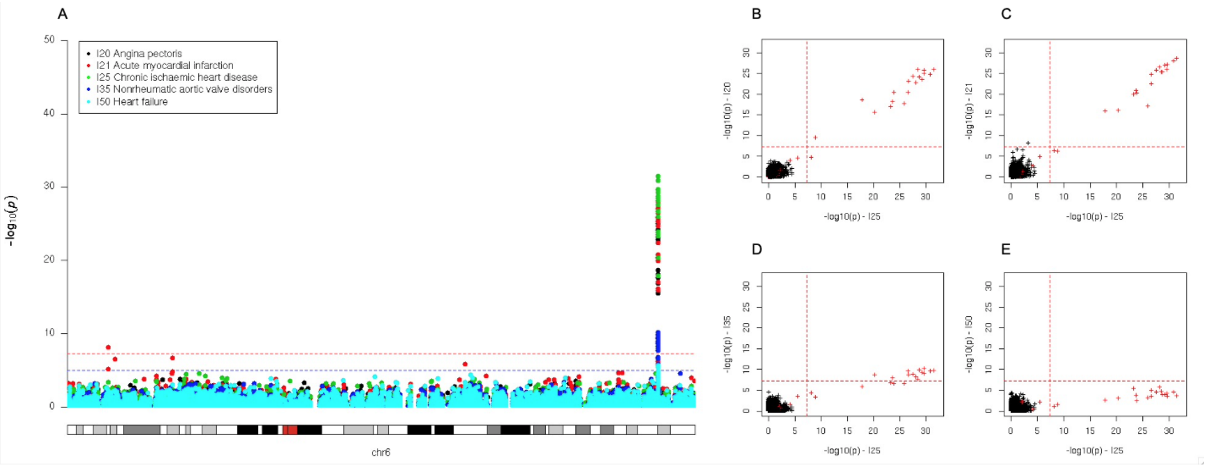

Supplementary Figure 8: *Comparison of association signal strength for heart related ICD10 codes at the LPA gene – relating to Figure 3. A: Overlaid manhattan plot from chromosome 6 including 5 heart related ICD10 code based case/control tests, the colour of points and legend indicate the ICD10 code. B: Minus log10 p values for ICD10 code I25 against code I20. C: Minus log10 p values for ICD10 code I25 against code I21. D: Minus log10 p values for ICD10 code I25 against code I35. E: Minus log10 p values for ICD10 code I25 against code I50.*

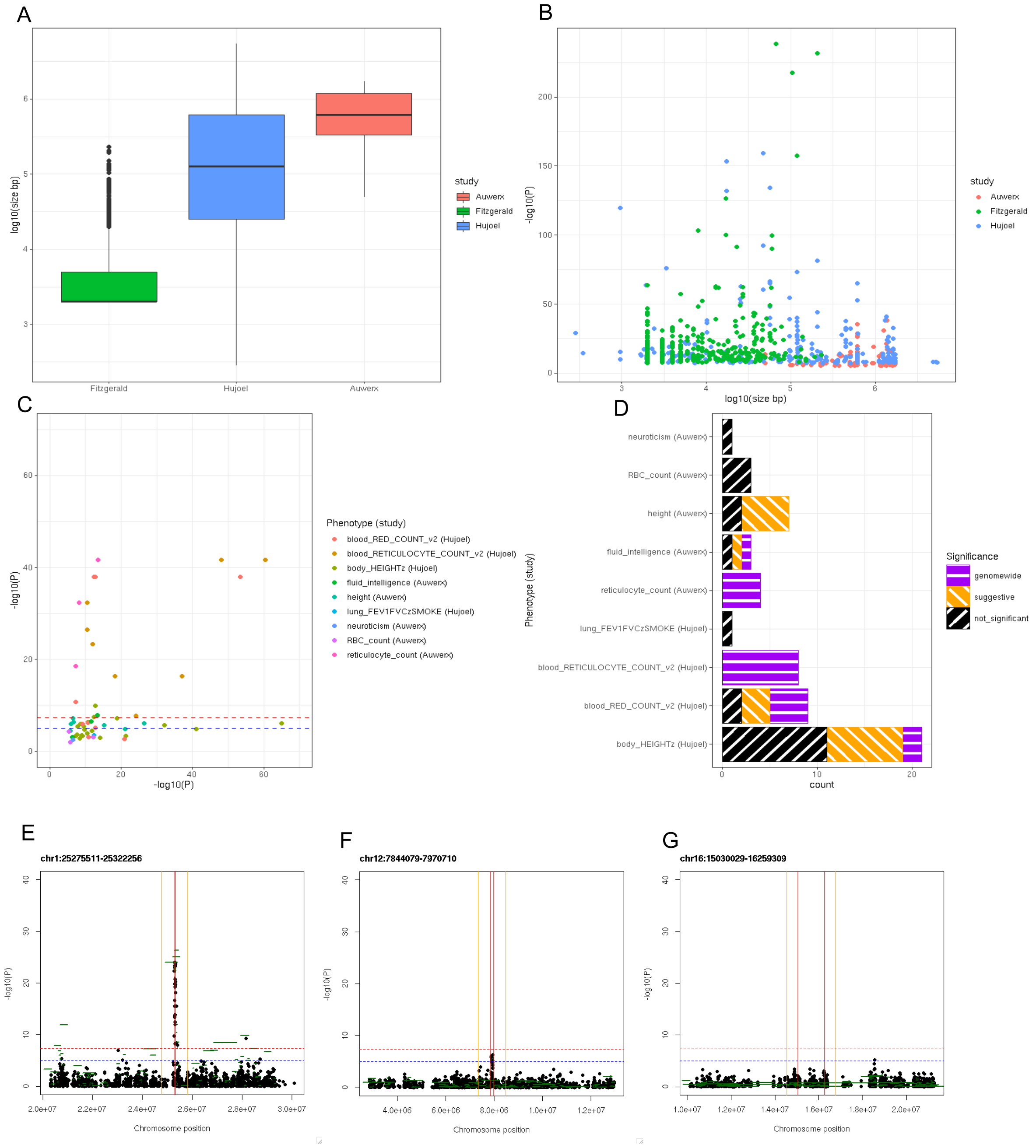

Supplementary Figure 9: *Summary of comparison into CNV association results between 2 SNP based CNV association studies (Auwerx et al[56] and Hujoel et al[57]) and CNest (Fitzgerald – this study) – relating to STAR Methods (comparison to previous association studies). A: Boxplot plots showing the log10 of the size (number of bases) for all fine mapped association regions with Auwerx in red, Hujoel in blue and Fitzgerald in green. B: The log10 of the size of all association regions against the -log10 p value for each of the 3 studies (Auwerx in red, Hujoel in blue and Fitzgerald in green). C: The -log10 p-value for the most significant exon level signal from CNest (Fitzgerald) against the -log10 p-value from each of the 2 SNP based CNV association studies across the 9 different traits, the color of point and legend indicate the trait and study. D: Stacked barplots showing the number of associations from the 2 SNP base studies and traits that show genomewide (purple), suggestive (orange) or no significant (black) association from exon level CNest association, the y-axis labels indicate the study and trait. E: Association region for reticulocyte count that shows genome wide significance in CNest results. F: Association region for height that shows suggestive significance in CNest results. G: Association region for the FEV/FEC score that shows no significance in CNest results.*

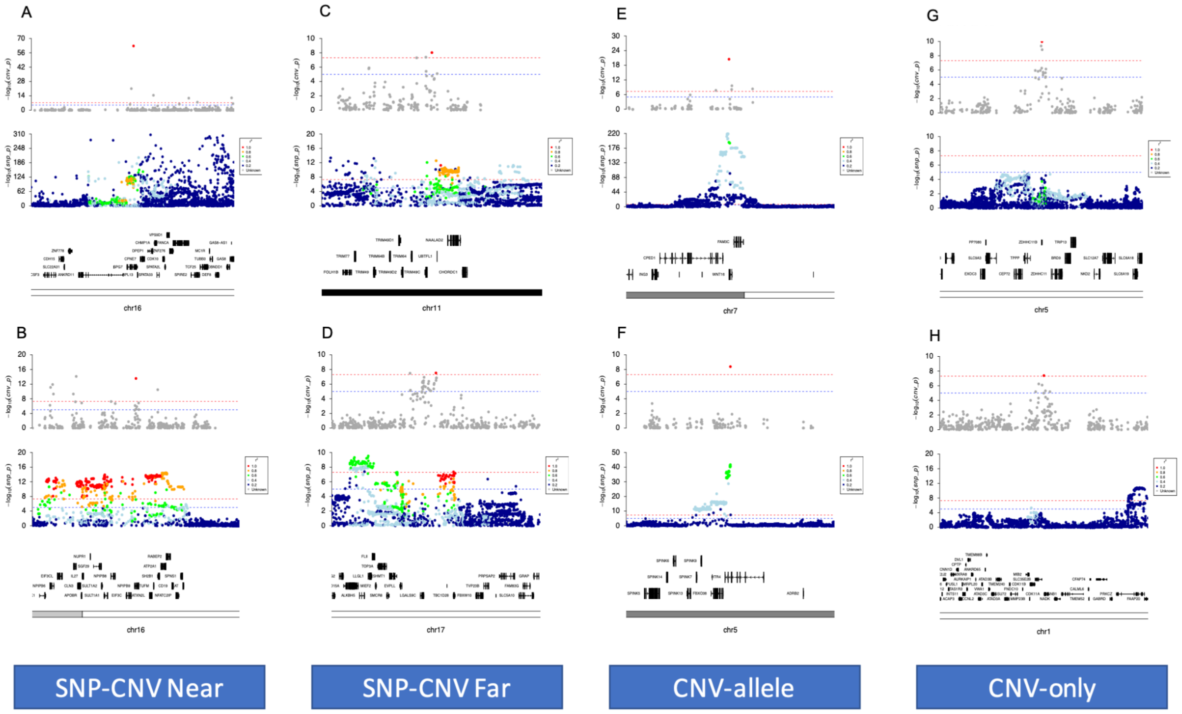

Supplementary Figure 10*: Locus zoom plots showing some CNV association categories – relating to Figure 4. A: SNP-CNV near discovery for hair colour involving exons 7-10 of the SPG7 gene. B: SNP-CNV near discovery for alcohol consumption at a 0.8MB region containing 4 fine mapped CNV regions involving the NPIPB6, NPIPB7, NPIPB9 and SH2B1 genes. C: SNP-CNV far discovery for hair colour at the TRIM49C with tagging SNPs downstream at UBTFL1 or NAALAD2 genes. D: SNP-CNV far discovery for standing height at a 12.6KB region including the EVPLL and LGALS9C genes. E: CNV-allele discovery for heel bone density at the WNT16 gene. F: CNV-allele discovery for the FEV/FEC ratio involving exon 1 of the HTR4 gene. G: CNV-only association for the FEV/FEC ratio on chromosome 5 at the ZDHHC11B gene. H: CNV-only discovery for standing height including several genes that are pulled up towards suggestive genome wide significance with a single exon signal that passes genome wide significance within the CDK11A gene.*

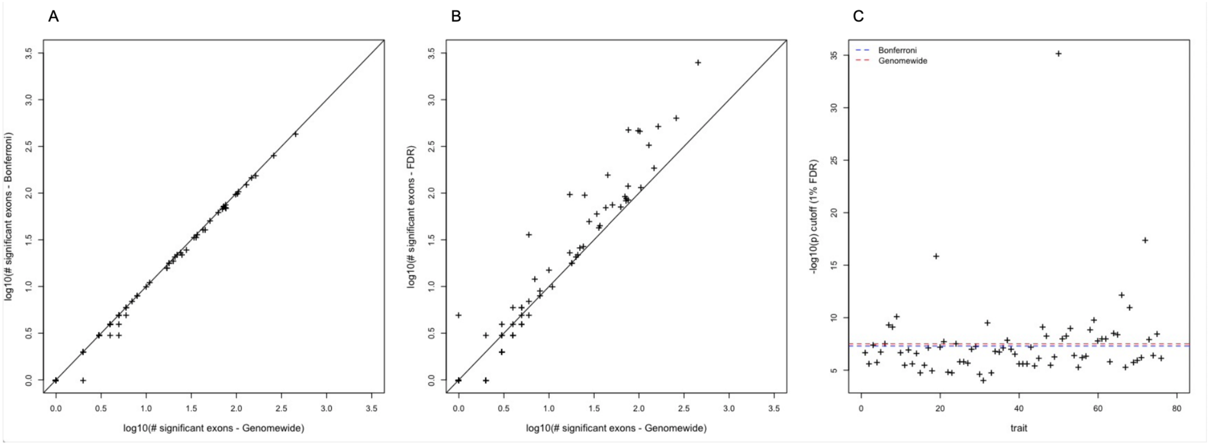

Supplementary Figure 11: *Comparison of significance level approaches – relating to STAR Methods (definition of the association significance threshold). A: log10 of the number of significant exon level signals per trait using the genome wide 5e-08 cut-off vs. a strict Bonferroni cut-off. B: log10 of the number of significant exon level signals per trait using the genome wide 5e-08 cut-off vs. a 1% FDR cut-off. C: The -log10 significance level at a 1% FDR cut-off for all traits tested. Red dashed line indicates the Genomewide 5e-08 cut-off and the blue dashed line shows the Bonferroni cut-off.*

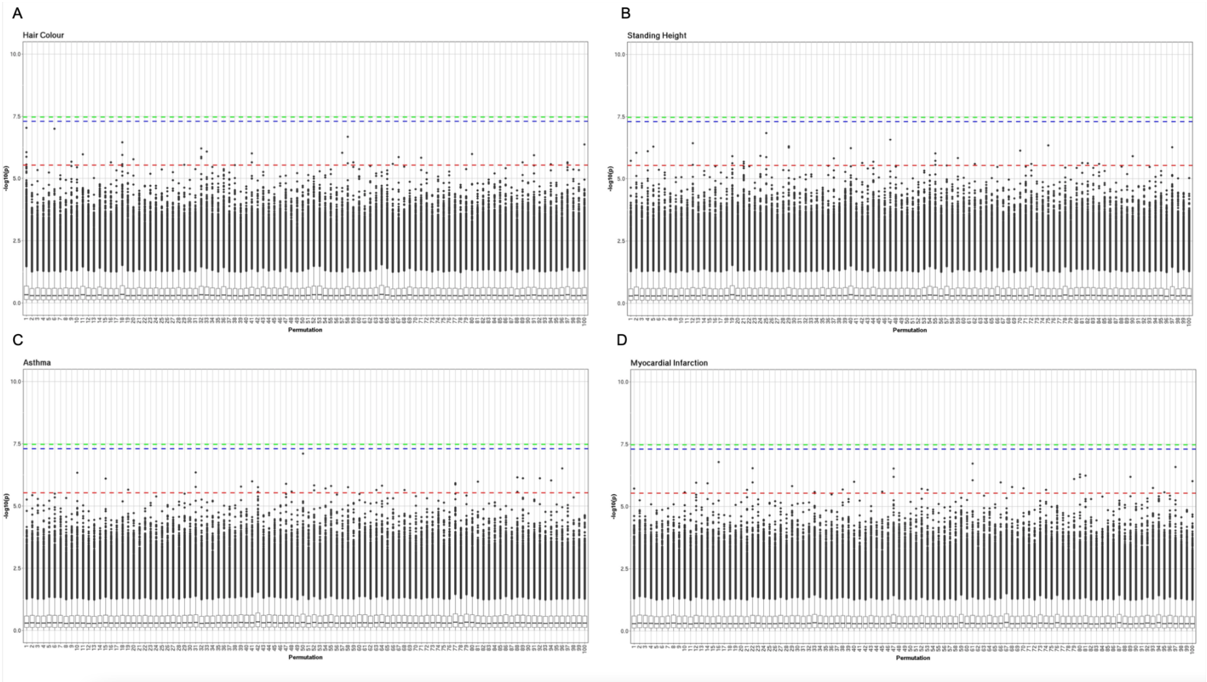

Supplementary Figure 12: *Permutation tests for the 4 main traits – relating to STAR Methods (definition of the association significance threshold). All tests were performed using a 100 different random ordering of the trait or case labels followed by association testing genomewide. A: Genomewide tests across 100 differently permuted traits for hair colour. B: Genomewide tests across 100 differently permuted traits for standing height. C: Genomewide tests across 100 differently permuted case labels for asthma. D: Genomewide tests across 100 differently permuted case labels for myocardial infarction.*

Supplementary data 1 – relating to Figure 2 (CNwas results on UK Biobank main traits)

Example - Hair Colour:
For hair colour (**Figure 2A**) after fine mapping we detect 30 copy number variable regions that pass genome wide significance, the majority of which (20/30) have been found to be associated with pigmentation by previous SNP GWAS (**Supplementary Table S4**). A particularly strong signal is found in a region containing the *OCA2* and *HERC2* genes with a -log10 P > 230. Both OCA2 and HERC2 are well known genes involved in pigmentation in both humans and mice and have been shown to be involved in iris, skin and hair pigmentation in human[117–120]. Pathogenic variation in *OCA2* including CNVs are known to be a leading cause of the rare genetic disorder oculocutaneous albinism and an increased susceptibility to melanoma[121–123]. The strongest signal and lead exon (**Figure 2E**) around the fine mapped CNV region is found in the hect domain and RCC1-like domain 2 (*HERC2*) which has been well described by multiple studies and has a strong association to pigmentation of the eyes, hair and skin[96,117,124]. Some of the novel associations we discover in relation to hair colour (**Supplementary Table S4**) include signals around 15q11.2, including several of the *GOLGA* genes which have functions relating to membrane traffic and Golgi structure; however, the precise function is unclear. Human chromosome 15 contains multiple copies of the GOLGA core elements close to the evolutionary conserved chromosome 15 low copy repeat (LCR15) duplicons[125] in primates at which several structural rearrangements break points have been described and linked to disorders and structural abnormalities such as Prader-Willi and Angelman syndromes[126]. Additionally, downstream *GOLGA* genes also detected here (*GOLGA8F*, *GOLGA8G* and *GOLGA8M*) have been linked to hair colour in previous SNP GWAS studies and from analysis of the UK Biobank[127].

Example - Standing Height:
After fine mapping we discovered 45 distinct regions associated with standing height encompassing between 1 and 2 genes (**Figure 2B**). The majority of these regions (27/45) contained at least one gene that had previously been associated with height from SNP GWAS (**Supplementary Table S5**) however with little to no evidence of CNV associations. The strongest signal outside of the HLA was seen in a region downstream of *ADAMTSL3* at 15q25.2, including the *UBE2Q2L* and *GOLGA6L4* genes and which is enriched for segmental duplications[128]. There is a single exon signal at exon 15 of the *ADAMTSL3* gene; *ADAMTSL3* has previous evidence from multiple SNP GWAS studies of being associated with height[129–132] and with certain neurological disorders such as schizophrenia[133] and bipolar disorders[134] acting under a proposed alternative splicing mechanism[135]. CNV at *ADAMTSL3* has yet to be described in relation to human height and interestingly this region contains multiple different CNV events varying in size all with strong association signals to height (**Figure 2F**). One for these CNVs overlaps exons 28-30 of *ADAMTSL3* which would likely result in truncation of the PLAC (protease and lacunin) domain[136]. Strong human height CNV association signals are observed in the segmental duplication rich region downstream of *ADAMTSL3* including exons within the *UBE2Q2L* and *GOLGA6L4* genes both of which have been linked to neurological disorders by previous work[137,138] with *UBE2Q2L* also having been specifically linked with human height[92,131]. Recurrent deletions and duplications at 15q25.2 have been described in relation to rare disease including neurological traits[139] however have not yet been described as a hotspot for structural rearrangements associated with common human traits such as height. We also found additional novel regions with no evidence of prior association to height (**Supplementary Table S5**), including one region at the Neuroblastoma BreakPoint Family *NBPF1* gene involving the

highly copy number variable *DUF1220* domain[140] which have be previously associated in a dose-dependent manner with important human traits such as microcephaly and macrocephaly, brain size and neurological disorders[19,141,142].

Example - Asthma:
For Asthma we discover 18 fine mapped CNV regions (**Figure 2C**). Strong CNV association signal was found around a region containing the 3 genes *CHROMR, PRKRA* and *PJVK* and upstream of *TTN* (**Figure 2G**). None of the 3 genes have previous evidence of a link to asthma however both *PRKRA* and *PJVK* have been found to be associated with lung functions such as vital capacity and forced expiratory volume (FEV) in a recent study[91]. In contrast the cholesterol induced regulator of metabolism RNA *CHROMR* has no previous association to asthma and its precise function is poorly understood. Unsurprisingly the strongest signal for asthma is found in the HLA with the lead signal specifically restricted to the *HLA-DQA2* gene which has strong prior associations to asthma and hay fever all based on intergenic SNPs from multiple SNP based GWAS investigations[143–147]. Some of our novel CNV associations for asthma (**Supplementary Table S6**) include signals in genes, *TAP2* and *STARD3NL*, which have not been linked with asthma by previous SNP GWAS studies but have evidence of association to certain other respiratory diseases[148,149]. A recent study using a different approach for CNV association testing from WES in the UKBB and specifically focussed on Asthma have rediscovered many of the associations we have made here[103].

Example - myocardial infarction:
For acute myocardial infarction (MI) we discover 26 fine mapped associations (**Figure 2D**). The strongest signal for MI is found within the *LPA* gene and this signal is found consistently across most heart related traits that we have tested in the UK Biobank. The *LPA* gene encodes a substantial portion of lipoprotein(a) and has been linked to numerous heart related diseases including coronary artery disease (CAD), aortic atherosclerosis and MI[150–152]. Changes in dosage of the *LPA* gene, specifically the KIV-2 copy number alteration, has been previously linked to changes in lipoprotein(a) levels and a modified risk of heart disease (CAD)[153–156]. This analysis of CNV association across a large cohort provides important additional information and allows a detailed estimate of the effect size for differences in *LPA* copy number in relation to the risk of MI. Interestingly we detect multiple different sized CNV events that hit the *LPA* gene (**Figure 2G**) but also include other coding regions with the lead exonic signal always restricted solely to the *LPA* gene. To our knowledge, only 2/26 fine mapped CNV associations (*LPA* and *BMP1*) have a direct association to MI from previous SNP GWAS testing[151,157] however a large fraction of the remaining regions have prior associations to other important heart related traits or cardiac disease risk factors (**Supplementary Table S7)**. For example, the *TM2D1* gene that has prior association to electrocardiography[158] and the structure of the left cardiac ventricle[159]; the *DPP6* gene that has been associated with multiple heart related phenotypes including sudden cardiac arrest[160]; and genes associated with blood lipid level measurements such as *LCAT* and *RCAN1*[161,162].

Supplementary data 2 – relating to Figure 3 (CNwas results on UK Biobank ICD10 first occurrences fields)

E*xample - E80:* For disorders of porphyrin and bilirubin metabolism, we found multiple strong signals involving specific exons across UDP-glucuronosyltransferase genes (*UGT1A10*, 9, 8, 7, 6 and 4) (**Figure 3E**). Genetic variation of *UGT1A* genes has been associated with disorders of bilirubin metabolism including Gilbert's syndrome by multiple previous SNP GWAS

studies[163,164] with, for example, very strong association signal at *UGT1A10* for the intron variant rs6742078 (2_233763993_G_T)[165]. This specific SNP has also been linked to other lipid metabolism disorders such as Gallstones Disease (GSD)[166] and although studies looking at CNV burden analysis of lipid metabolism genes have shown a significant enrichment in GSD cases none of those associations could be attributed to any single gene[167]. Here we provide novel CNV associations at *UGT1A* genes with a direct link to bilirubin metabolism that could be an important risk factor for several lipid metabolism related disorders.

Example - D50: For iron deficiency anaemia we discovered two significantly associated loci on chromosome 7 (**Figure 3F**) one of which covers exons 4-6 of the cationic trypsinogene gene *PRSS1* that has been linked to chronic pancreatitis by multiple studies[168,169]. Autosomal dominant mutations in *PRSS1* are thought to be a leading cause of hereditary pancreatitis, a rare condition that results in recurrent inflammation of the pancreas, and an increased risk of pancreatic cancer[170]. As such *PRSS1* is regularly tested in patients with suspected hereditary pancreatitis[171] however the *PRSS1* gene contains multiple known variants, including copy number changes, often with unknown clinical importance[172]. Iron metabolism and pancreatic function are closely related processes[173] with evidence that pancreatic enzyme levels influence the efficiency of iron absorption[174]. Here we provide a link between the copy number at exons 4-6 of the *PRSS1* gene with the ICD10 code D50 relating to iron deficiency anaemia that may be a result of pancreatic dysfunction.

Example - M10: For Musculoskeletal disorders we discovered 17 fine mapped association loci across 4 different traits including one location at 4p16.1 at exon 3 of the *SLC2A9* gene that was associated with ICD10 code M10: gout (**Figure 3G**). Gout is a swelling of joints, normally in the feet, that is caused by hyperuricemia (an excess of uric acid in the blood) with mutations at *SLC2A9* having been found to be associated with serum urate concentrations and the onset of gout[175,176]. A non coding CNV near *SLC2A9* (integenic and approximately 200 kb upstream of the *SLC2A9* gene) has been described in association with serum uric acid levels[177] however CNVs in coding regions of the *SLC2A9* have not yet been discovered in relation to uric acid level or with a direct association to gout. Here we provide a novel CNV association result at exon 3 of the *SLC2A9* gene with a direct association to gout from the UK Biobank.

Example - O36: For Pregnancy childbirth and the puerperium we discovered fine mapped CNV associations against code O36: maternal care for known or suspected foetal problems at 1p36.11 including the *RHD* and *RHCE* genes (**Figure 3H**). Variation and *RHD* gene deletion in the human Rh blood group system has been extensively studied in relation to pregnancy risk[178] where prior to the development of medical treatments, Rh-negative (D-negative) mothers were at significant risk of haemolytic disease of the newborn (HDN). It is still unclear what potential benefit the *RHD* gene deletion may have that merits its relatively high frequency in the human population[179]. Blood tests are normally carried out in D-negative expectant mothers to determine the Rh factor status of the child and direct treatment if using anti-D injection is required[180]. However variation in the less well understood Rh C and E alleles of *RHCE* is clinically relevant, influences the risk of HDN[181] and this association discovered in this study merits further investigation for this well understood risk factor for pregnancy.

Example - K74: For fibrosis and cirrhosis of liver we discovered a single CNV association at exon 3 of the *PNPLA3* gene (**Figure 3I**). Cirrhosis of the liver is a disorder in which the liver parenchyma is replaced with fibrous tissue and is often caused by alcoholism as well as hepatitis B and C infection[182,183]. The *PNPLA3* gene has been found to be associated with liver cirrhosis by multiple SNP GWAS studies[184,185] and although CNVs at *PNPLA3* has not been well

described or linked to Cirrhosis in the past it has been shown that transcriptional regulation of *PNPLA3* has an impact on liver disease with higher levels of *PNPLA3* mRNA in the cytoplasm being negatively associated with the severity of alcoholic fatty liver disease (NAFLD) in humans[186].

Example - heart related ICD10 codes: Across 5 heart related ICD10 codes (I20, I21, I25, I35 and I50) we found strong CNV association signals at the *LPA* gene with the exception of I50: heart failure (**Supplementary Figure S8).** When comparing signal strength between the 5 heart related ICD10 codes we observe a clear sample size effect with the tests showing the stronger signals tending to have larger number of cases (**Supplementary Figure S8 B-E**). The five ICD10 codes included were I25: chronic ischaemic heart disease (20,503 cases), I20: angina pectoris (10,117 cases), I21: acute myocardial infarction (3,698 cases), I35: nonheumatic aortic valve disorders (1,692 cases) and I50: heart failure (3,557 cases). Although there is heterogeneity between the effect sizes for these different ICD codes, ranging from -0.39 for I25 to -1.41 for I35, they are similar for the 3 codes I20, I25 and I50 (-0.42, -0.39 and -0.38 respectively), suggesting that this association may become significant for I50 with increased sample sizes. CNV association at *LPA* is a major feature in all heart related phenotypes we have tested in the UK Biobank providing further evidence that changes in dosage of *LPA* is a significant risk factor for heart disease in humans.

Supplementary data 3 – relating to Figure 4 (combined CNV and SNP based associations)

For standing height we show clear complementary signals at the ACAN gene for SNP based and CNV based association results (**Figure 4A**), an example of the SNP-CNV near class with the CNV signal being well tagged by 30 SNPs within the gene body between exons 6 to 12. The CNV signal is restricted to exon 12 that encodes the chondroitin sulfate attachment (CS) domain[187] which is important for aggregation with hyaluronan resulting in a strong negative charge that gives rise to load-bearing properties of cartilage[188]. Mutations in *ACAN* have been studied in relation to both syndromic and nonsyndromic human traits with a number of different impactful variants having been discovered[130,189,190]. Earlier studies found *ACAN* to be a strong candidate for autosomal dominant disorders such as spondyloepiphyseal dysplasia Kimberley type (SEDK) and early-onset osteoarthritis (OA) from genetic linkage analysis and mouse models of chondrodysplasia[187] however, heterozygous mutations in *ACAN* display highly variable nonsyndromic phenotypes including short stature, early onset osteoarthritis and mild dysmorphic features[191]. In this case, although the CNV is both well tagged and close to the SNP associations, the CNV directly suggests the functional variant of these SNPs is the deletion of this exon implying that haploinsufficiency of the *ACAN* gene is the main mechanism underlying these associations.

A *SNP-CNV far* example is shown in **Figure 4B**, being a 60KB region on chromosome 6 that is associated with the lung function measure FEV/FEC ratio including the *STK19, C4A, C4B* and *CYP21A2* genes with the lead exon CNV signal encompassing exons 26-30 of the *C4A* gene (**Figure 4B**). Interestingly, there are 208 tagging SNPs that pass genome wide significance for FEV/FEC ratio however none of these SNPs are located closest to either of the *C4* genes (*C4A* or *C4B).* The C4 genes encode an important part of the immune complement system and deficiencies (including CNV) at *C4* genes have been strongly associated with immune disorders such as Systemic Lupus Erythematosus[192,193]. The *C4A* and *C4B* genes encode different components of the highly polymorphic C4 complement protein and can be distinguished from each other by four specific amino acids at positions 1101–1106[194]. Due to

high sequence similarity the total copy number of *C4* can be defined as the sum between *C4A* and *C4B*[195], however both *C4A* and *C4B* are multiallelic CNV locations displaying common differences in copy number with *C4A* ranging between 0 to 5 and *C4B* between 0 to 4 copies[196]. The CNVs at *C4* has not been linked previously with lung function; however, a recent study into chronic obstructive pulmonary disease (COPD) in the Korea Associated Resource cohort has investigated genome wide SNP interactions mapped to *C4B* in relation COPD and the FEV/FEC ratio measure[197]. We provide new evidence for the role of *C4* CNV in lung function as measured by the FEV/FEC ratio in the UK Biobank.

SUPPLEMENTAL REFERENCES

117. Sulem, P., Gudbjartsson, D.F., Stacey, S.N., Helgason, A., Rafnar, T., Jakobsdottir, M., Steinberg, S., Gudjonsson, S.A., Palsson, A., Thorleifsson, G., et al. (2008). Two newly identified genetic determinants of pigmentation in Europeans. Nat. Genet. *40*, 835-837.

118. Zhang, M., Song, F., Liang, L., Nan, H., Zhang, J., Liu, H., Wang, L.-E., Wei, Q., Lee, J.E., Amos, C.I., et al. (2013). Genome-wide association studies identify several new loci associated with pigmentation traits and skin cancer risk in European Americans. Hum. Mol. Genet. *22*, 2948-2959.

119. Han, J., Kraft, P., Nan, H., Guo, Q., Chen, C., Qureshi, A., Hankinson, S.E., Hu, F.B., Duffy, D.L., Zhao, Z.Z., et al. (2008). A genome-wide association study identifies novel alleles associated with hair color and skin pigmentation. PLoS Genet. *4*, e1000074.

120. Lin, B.D., Willemsen, G., Abdellaoui, A., Bartels, M., Ehli, E.A., Davies, G.E., Boomsma, D.I., and Hottenga, J.J. (2016). The genetic overlap between hair and eye color. Twin Res. Hum. Genet. *19*, 595-599.

121. Eriksson, N., Macpherson, J.M., Tung, J.Y., Hon, L.S., Naughton, B., Saxonov, S., Avey, L., Wojcicki, A., Pe'er, I., and Mountain, J. (2010). Web-based, participant-driven studies yield novel genetic associations for common traits. PLoS Genet. *6*, e1000993.

122. Akiyama, M., Ishigaki, K., Sakaue, S., Momozawa, Y., Horikoshi, M., Hirata, M., Matsuda, K., Ikegawa, S., Takahashi, A., Kanai, M., et al. (2019). Characterizing rare and low-frequency height-associated variants in the Japanese population. Nat. Commun. *10*, 4393.

123. Wood, A.R., Esko, T., Yang, J., Vedantam, S., Pers, T.H., Gustafsson, S., Chu, A.Y., Estrada, K., Luan J., Kutalik, Z., et al. (2014). Defining the role of common variation in the genomic and biological architecture of adult human height. Nat. Genet. *46*, 1173-1186.

124. He, M., Xu, M., Zhang, B., Liang, J., Chen, P., Lee, J.-Y., Johnson, T.A., Li, H., Yang, X., Dai, J., et al. (2015). Meta-analysis of genome-wide association studies of adult height in East Asians identifies 17 novel loci. Hum. Mol. Genet. *24*, 1791-1800.

125. Tachmazidou, I., Süveges D., Min, J.L., Ritchie, G.R.S., Steinberg, J., Walter, K., Iotchkova, V., Schwartzentruber, J., Huang, J., Memari, Y., et al. (2017). Whole-genome sequencing coupled to imputation discovers genetic signals for anthropometric traits. Am. J. Hum. Genet. *100*, 865-884.

126. Lango Allen, H., Estrada, K., Lettre, G., Berndt, S.I., Weedon, M.N., Rivadeneira, F., Willer, C.J., Jackson, A.U., Vedantam, S., Raychaudhuri, S., et al. (2010). Hundreds of variants clustered in genomic loci and biological pathways affect human height. Nature *467*, 832-838.

127. Gudbjartsson, D.F., Walters, G.B., Thorleifsson, G., Stefansson, H., Halldorsson, B.V., Zusmanovich, P., Sulem, P., Thorlacius, S., Gylfason, A., Steinberg, S., et al. (2008). Many sequence variants affecting diversity of adult human height. Nat. Genet. *40*, 609-615.

128. Rüeger S., McDaid, A., and Kutalik, Z. (2018). Evaluation and application of summary statistic imputation to discover new height-associated loci. PLoS Genet. *14*, e1007371,

129. Wojcik, G.L., Graff, M., Nishimura, K.K., Tao, R., Haessler, J., Gignoux, C.R., Highland, H.M., Patel, Y.M., Sorokin, E.P., Avery, C.L., et al. (2019). Genetic analyses of diverse populations improves discovery for complex traits. Nature *570*, 514-518.

130. Nagy, R., Boutin, T.S., Marten, J., Huffman, J.E., Kerr, S.M., Campbell, A., Evenden, L., Gibson, J., Amador, C., Howard, D.M., et al. (2017). Exploration of haplotype research consortium imputation for genome-wide association studies in 20, 032 Generation Scotland participants. Genome Med. *9*, 23.

131. Ramasamy, A., Kuokkanen, M., Vedantam, S., Gajdos, Z.K., Couto Alves, A., Lyon, H.N., Ferreira, M.A.R., Strachan, D.P., Zhao, J.H., Abramson, M.J., et al. (2012). Genome-wide association studies of asthma in population-based cohorts confirm known and suggested loci and identify an additional association near HLA. PLoS One *7*, e44008.

132. Shrine, N., Portelli, M.A., John, C., Soler Artigas, M., Bennett, N., Hall, R., Lewis, J., Henry, A.P., Billington, C.K., Ahmad, A., et al. (2019). Moderate-to-severe asthma in individuals of European ancestry: a genome-wide association study. Lancet Respir. Med. *7*, 20-34.

133. Pividori, M., Schoettler, N., Nicolae, D.L., Ober, C., and Im, H.K. (2019). Shared and distinct genetic risk factors for childhood-onset and adult-onset asthma: genome-wide and transcriptome-wide studies. Lancet Respir. Med. *7*, 509-522.

134. Zhu, Z., Zhu, X., Liu, C.-L., Shi, H., Shen, S., Yang, Y., Hasegawa, K., Camargo, C.A., Jr, and Liang, L. (2019). Shared genetics of asthma and mental health disorders: a large-scale genome-wide cross-trait analysis. Eur. Respir. J. *54*, 1901507.

135. Klimentidis, Y.C., Raichlen, D.A., Bea, J., Garcia, D.O., Wineinger, N.E., Mandarino, L.J., Alexander, G.E., Chen, Z., and Going, S.B. (2018). Genome-wide association study of habitual physical activity in over 377, 000 UK Biobank participants identifies multiple variants including CADM2 and APOE. Int. J. Obes. *42*, 1161-1176.

136. Hartiala, J.A., Han, Y., Jia, Q., Hilser, J.R., Huang, P., Gukasyan, J., Schwartzman, W.S., Cai, Z., Biswas, S., Trégouët D.A., et al. (2021). Genome-wide analysis identifies novel susceptibility loci for myocardial infarction. Eur. Heart J. *42*, 919-933.

137. Richardson, T.G., Sanderson, E., Elsworth, B., Tilling, K., and Davey Smith, G. (2020). Use of genetic variation to separate the effects of early and later life adiposity on disease risk: mendelian randomisation study. BMJ *369*, m1203.
